# The B-Cell Specific Transcription Factor, Oct-2, Promotes Epstein-Barr Virus Latency by Inhibiting the Viral Immediate-Early Protein, BZLF1

**DOI:** 10.1371/journal.ppat.1002516

**Published:** 2012-02-09

**Authors:** Amanda R. Robinson, Swee Sen Kwek, Shannon C. Kenney

**Affiliations:** 1 Department of Oncology, McArdle Laboratory for Cancer Research , University of Wisconsin School of Medicine and Public Health, Madison, Wisconsin, United States of America; 2 Department of Cellular and Molecular Biology, University of Wisconsin School of Medicine and Public Health, Madison, Wisconsin, United States of America; 3 Department of Medicine, University of Wisconsin School of Medicine and Public Health, Madison, Wisconsin, United States of America; Wistar Institute, United States of America

## Abstract

The Epstein-Barr virus (EBV) latent-lytic switch is mediated by the BZLF1 immediate-early protein. EBV is normally latent in memory B cells, but cellular factors which promote viral latency specifically in B cells have not been identified. In this report, we demonstrate that the B-cell specific transcription factor, Oct-2, inhibits the function of the viral immediate-early protein, BZLF1, and prevents lytic viral reactivation. Co-transfected Oct-2 reduces the ability of BZLF1 to activate lytic gene expression in two different latently infected nasopharyngeal carcinoma cell lines. Furthermore, Oct-2 inhibits BZLF1 activation of lytic EBV promoters in reporter gene assays, and attenuates BZLF1 binding to lytic viral promoters *in vivo*. Oct-2 interacts directly with BZLF1, and this interaction requires the DNA-binding/dimerization domain of BZLF1 and the POU domain of Oct-2. An Oct-2 mutant (Δ262–302) deficient for interaction with BZLF1 is unable to inhibit BZLF1-mediated lytic reactivation. However, an Oct-2 mutant defective for DNA-binding (Q221A) retains the ability to inhibit BZLF1 transcriptional effects and DNA-binding. Importantly, shRNA-mediated knockdown of endogenous Oct-2 expression in several EBV-positive Burkitt lymphoma and lymphoblastoid cell lines increases the level of lytic EBV gene expression, while decreasing EBNA1 expression. Moreover, treatments which induce EBV lytic reactivation, such as anti-IgG cross-linking and chemical inducers, also decrease the level of Oct-2 protein expression at the transcriptional level. We conclude that Oct-2 potentiates establishment of EBV latency in B cells.

## Author Summary

Epstein-Barr virus (EBV) is a human herpesvirus associated with B-cell malignancies. EBV infection of cells can result in either lytic replication or latency. Memory B cells are the primary site of EBV latency within the human host, while oropharyngeal epithelial cells support the lytic form of infection. However, the cellular mechanism(s) that enable EBV to establish viral latency in a B-cell specific manner are not currently understood. In this report, we show that the B-cell specific cellular transcription factor, Oct-2, promotes viral latency by inhibiting the lytic form of infection. We find that Oct-2 interacts directly with the EBV immediate-early protein, BZLF1, and abrogates its ability to activate lytic viral gene transcription through protein-protein interactions off the DNA. Furthermore, knockdown of endogenous Oct-2 expression in several latently-infected Burkitt lymphoma B-cell lines increases EBV lytic protein expression. In addition, we show that certain stimuli which can prompt lytic EBV reactivation in B cells also decrease expression of endogenous Oct-2. Our results suggest that the cellular transcription factor, Oct-2, promotes EBV latency in a B-cell dependent manner.

## Introduction

Epstein-Barr virus (EBV) is the causative agent of infectious mononucleosis and is widely prevalent within the human population, infecting greater than 90% of all individuals [1], [2]. The virus is also associated with several different diseases, including, but not limited to, Burkitt lymphoma (BL), Hodgkin disease (HD), nasopharyngeal carcinoma (NPC), T/NK lymphoma, and gastric carcinoma [1], [3]. Like all herpesviruses, EBV infection of cells can result in either lytic replication or latency. In the normal host, memory B cells serve as the primary reservoir of latent EBV infection, while oropharyngeal epithelial cells support the lytic form of infection [1], [2], [4]–[8]. EBV can also be reactivated to the lytic form when infected memory B cells, stimulated by antigen, differentiate into plasma cells [9]. While both the latent and lytic forms of infection are essential for the long-term success of EBV, the specific cellular factors that determine the very different outcomes following EBV infection in B cells versus epithelial cells remain poorly defined.

Several different types of EBV latency have been described [2]. Type III latency is characterized by the expression of all nine EBV latent proteins (EBNA1, EBNA2, EBNA3A-C, EBNA-LP, LMP1, LMP2A-B), and occurs in EBV-transformed lymphoblastoid B-cell lines (LCLs), as well as some BL lines in culture [10], [11]. In cells with the most restricted form of viral latency, known as type I (characteristic of BL tumors *in vivo*), the only viral protein expressed is EBNA1. The EBNA1 transcript is derived from the viral Q promoter (Qp) in cells with type I latency, versus the C promoter (Cp) in cells with type III latency [12]. Cellular factors that determine the type of viral latency are not currently well understood.

EBV lytic reactivation can be initiated by expression of either the BZLF1 (Z, Zta, ZEBRA, EB1) or BRLF1 (R, Rta) immediate-early (IE) viral gene products [13]–[19]. The BZLF1 and BRLF1 proteins are transcription factors which activate each other's promoters, as well as their own promoters [18]–[26]. This enables the virus to amplify weak lytic induction stimuli. BZLF1 binds to AP1 DNA sites, as well as AP1-like sites termed ZREs (BZLF1-responsive elements), that are found in many early lytic EBV viral promoters, including the BZLF1 and BRLF1 promoters [23], [24]. Interestingly, CpG methylation of many promoters containing ZREs results in increased BZLF1 binding and transactivation of these promoters [25], [27], [28]. BRLF1 binds to a GC-rich motif and activates some lytic viral promoters through a direct DNA-binding mechanism [29]–[33], although BRLF1 activation of some viral promoters (including the BZLF1 promoter) occurs though indirect mechanisms [20]. Together, BZLF1 and BRLF1 cooperatively (and for some genes synergistically) activate expression of the entire lytic viral gene program, leading to productive lytic viral replication [21], [23], [30], [33]–[39]. Since both BZLF1 and BRLF1 are required to activate many of the lytic viral genes, BZLF1 must first turn on BRLF1 expression (and vice versa) to successfully induce full lytic reactivation in the context of the intact viral genome.

Cellular transcription factors that regulate the activity of the BZLF1 and/or BRLF1 promoters play a major role in determining the level of lytic gene expression [1], [2]. For example, ZEB1 promotes viral latency by binding directly to, and inhibiting transcription of, the BZLF1 promoter [40]–[43]. Conversely, the activated form of XBP1 (XBP1-s), which is expressed during plasma cell differentiation, activates both the BZLF1 and BRLF1 promoters, thereby intiating the viral lytic cycle [44], [45]. Stimulation of the B-cell receptor with anti-IgG induces a signal transduction cascade that results in lytic reactivation in BL lines, and this effect is at least partially mediated through phosphatidylinositol 3-kinase/Ca^2+^ -induced dephosphorylation of the MEF2D protein (converting it from a negative to positive regulator of the BZLF1 promoter) [46]. In addition, the TGF-β cytokine induces lytic reactivation in some BL lines by promoting SMAD2/3/4-mediated activation of the BZLF1 promoter [47].

Cellular factors that regulate the function of the BZLF1 and/or BRLF1 proteins also influence the abililty of the virus to reactivate [36], [48]–[54]. For example, we recently demonstrated that the POU domain transcription factor, Oct-1, enhances BRLF1 transcriptional function and DNA-binding through a direct protein-protein interaction between Oct-1 and BRLF1 [55]. In addition, the TORC2 protein promotes BZLF1 function, while the p65 component of NFκB inhibits BZLF1 function, through direct protein-protein interactions with the BZLF1 protein [51], [54]. However, cellular proteins that promote viral latency in a B-cell specific manner have not yet been identified.

The POU domain transcription factors contain a conserved POU domain which mediates protein-protein interactions and binds to the octamer DNA motif (consensus site ATGCAAAT) [56]–[58]. In addition to its effect on EBV reactivation [55], the POU domain family member Oct-1 also promotes lytic gene expression of several other herpesviruses. The Kaposi Sarcoma Herpesvirus (KSHV) ORF50 (Rta) immediate-early protein interacts directly with Oct-1, and this interaction is required for ORF50 activation of the early lytic K-bZIP (K8) promoter, as well as its own promoter, in certain cell lines [59], [60]. In addition, Oct-1 interaction with the herpes simplex virus (HSV) and varicella zoster virus (VZV) encoded tegument proteins (VP16 and ORF10 respectively), is required for efficient activation of the HSV and VZV immediate-early promoters [61]–[65].

Oct-2, like Oct-1, is a member of the POU (Pit-Oct-Unc) domain family [66]–[69]. In contrast to the ubiquitously expressed Oct-1 protein, Oct-2 expression is restricted to B cells and neuronal cells [66]–[69]. As opposed to the effect of Oct-1, Oct-2 has been reported to inhibit lytic KSHV reactivation by competing for Oct-1 binding sites in the KSHV ORF50 promoter [70]. Furthermore, certain isoforms of Oct-2 (preferentially expressed in neuronal cells) promote HSV latency in neurons by binding to, and repressing, the ICP0 IE promoter [71].

Oct-2 can act as either a positive or negative regulator of transcription, depending upon its interaction with co-activators (such as Bob-1) [72], [73] versus co-repressors (such TLE1/2) [74]. In the case of EBV, the cellular Oct-2 transcription factor has been proposed to promote type I (versus type III) latency by binding to the FR repeat elements (in conjunction with TLE family members) and inhibiting the activity of the downstream Cp type III latency promoter [74], [75]. However, another report suggested that Oct-2 binding to the FR repeats enhances the activity of the Cp [72]. Furthermore, since the previous reports were based upon the results of reporter gene assays and over-expressed Oct-2, the effect of endogenous Oct-2 upon the regulation of EBV gene expression in the context of the intact viral genome has remained uncertain.

In this paper we have examined the hypothesis that the Oct-2 transcription factor promotes EBV latency in a B-cell specific manner. Oct-2 expression is decreased following B-cell differentiation into plasma cells [76], making it an attractive candidate to negatively regulate EBV lytic reactivation. To date, however, the role of Oct-2 in regulating EBV lytic gene expression has not been investigated. Here we show that Oct-2 inhibits EBV lytic reactivation by attenuating BZLF1 function. We find that Oct-2 directly interacts with BZLF1 *in vitro* and *in vivo*, and inhibits BZLF1 binding to EBV promoters *in vivo*. A DNA-binding defective Oct-2 mutant (Q221A) retains the ability to inhibit BZLF1 function. Furthermore, we show that shRNA-mediated knockdown of endogenous Oct-2 increases lytic protein expression (while decreasing EBNA1) in EBV-positive B-cell lines with either type I or type III latency. Finally, we show that two different lytic inducing stimuli (anti-IgG and 12-*O*-tetradecanoyl-phorbol-13-acetate (TPA)/sodium butyrate) also decrease expression of endogenous Oct-2 in EBV-infected B cells. Together, these results indicate that Oct-2 acts as a potent negative regulator of EBV lytic reactivation, and suggest a mechanism by which EBV latency is specifically promoted in B cells.

## Results

### Oct-2 inhibits BZLF1-mediated lytic viral reactivation

We recently reported that the ubiquitous Oct-1 transcription factor promotes lytic EBV reactivation [55]. To determine if the B-cell specific Oct-2 transcription factor has a similar effect, we transfected either a BZLF1 or BRLF1 expression vector, in the presence or absence of a co-transfected Oct-2 vector (expressing the major B-cell form of Oct-2, isoform 1), into latently infected EBV-positive HONE-Akata NPC cells. Immunoblot analysis was performed two days after transfection to assess the level of transfected BZLF1 or BRLF1 proteins, as well as their ability to induce expression of lytic EBV proteins from the endogenous viral genome.

**Figure 1:**
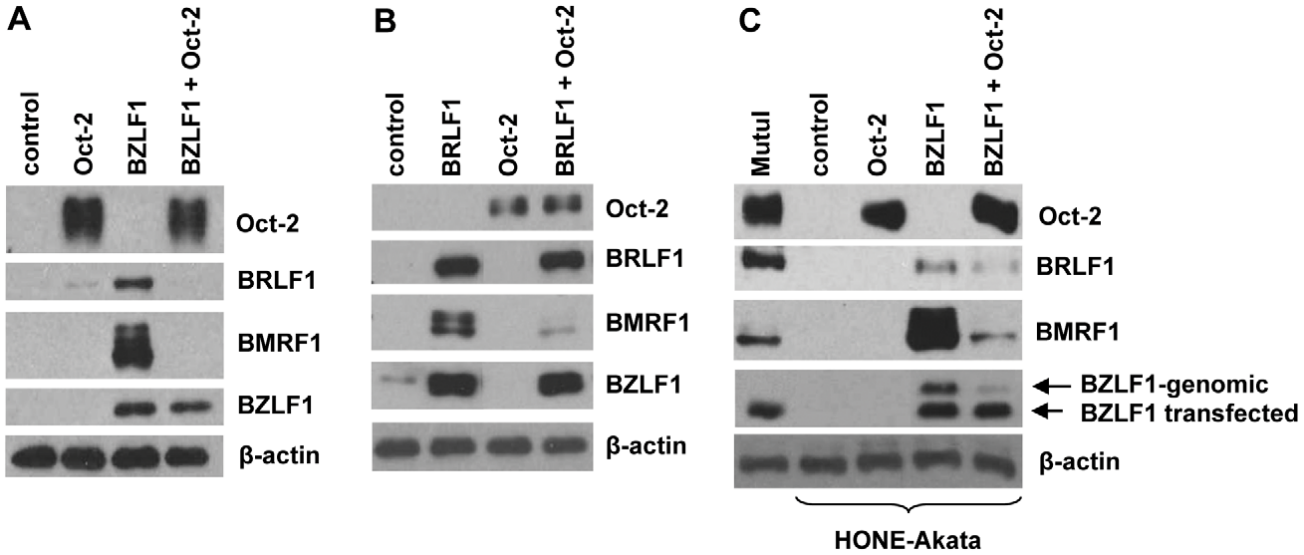
Oct-2 inhibits BZLF1-mediated lytic viral reactivation. (A) EBV-positive HONE-Akata cells were transfected with 5 ng BZLF1, 500 ng Oct-2, or control expression vectors as indicated. Immunoblot analysis was performed two days after transfection to compare the levels of transfected Oct-2 and BZLF1, as well as the lytic viral proteins BMRF1 and BRLF1 derived from the endogenous viral genome. β-actin expression was used as a loading control. (B) HONE-Akata cells were transfected with 5 ng BRLF1, 500 ng Oct-2, or control expression vectors as indicated. Immunoblot analysis was performed two days after transfection to compare the levels of transfected Oct-2 and BRLF1, as well as the lytic viral proteins BMRF1 and BZLF1 derived from the endogenous viral genome. β-actin expression was used as a loading control. (C) HONE-Akata cells were transfected with control vector or 50 ng Oct-2 expression vector (with or without 5 ng BZLF1) as indicated. Immunoblot analysis was performed two days after transfection to compare the levels of transfected and endogenous Oct-2 in HONE-Akata and MutuI cells, the levels of transfected BZLF1, and the lytic viral proteins, BZLF1 (genomic BZLF1), BRLF1, and BMRF1, derived from the endogenous viral genome. β-actin served as a loading control.

In contrast to the previously reported effect of Oct-1 [55], co-transfected Oct-2 greatly reduced BZLF1-mediated activation of the BRLF1 IE protein, as well as the early lytic BMRF1 protein (Figure 1A). In contrast, co-transfected Oct-2 did not affect the ability of BRLF1 to activate expression of BZLF1 from the endogenous viral genome; however, the ability of BRLF1 to activate expression of the early viral protein, BMRF1, from the endogenous viral genome was reduced (Figure 1B). Since the BMRF1 promoter is known to require both BZLF1 and BRLF1 function to be activated efficiently in the context of the intact viral genome [21], [34], these results are consistent with a model in which Oct-2 primarily inhibits BZLF1 (rather than BRLF1) function.

The experiments shown in Figures 1A and B (which used 500 ng of transfected Oct-2 expression vector per 12-well dish), resulted in Oct-2 expression levels greatly above that found in EBV-infected B cells (data not shown). To determine if Oct-2 can inhibit BZLF1 function when expressed at a level in HONE-Akata cells similar to that expressed in EBV-infected BL cells, we transfected different amounts of Oct-2 into HONE-Akata cells and performed western blots to compare the Oct-2 expression in the transfected epithelial cells versus the endogenous Oct-2 level in MutuI BL cells. These results indicated that 50 ng of transfected Oct-2 plasmid vector results in an Oct-2 protein level in HONE-Akata cells similar to the endogenous Oct-2 level in MutuI cells (Figure 1C). Importantly, this level of Oct-2 expression in HONE-Akata cells was sufficient to decrease BZLF1-mediated activation of its own promoter, as well as BMRF1 and BRLF1 (Figure 1C). Therefore, Oct-2 is able to inhibit lytic reactivation when expressed at physiologic levels.

### Oct-2 inhibits lytic EBV replication

**Figure 2:**
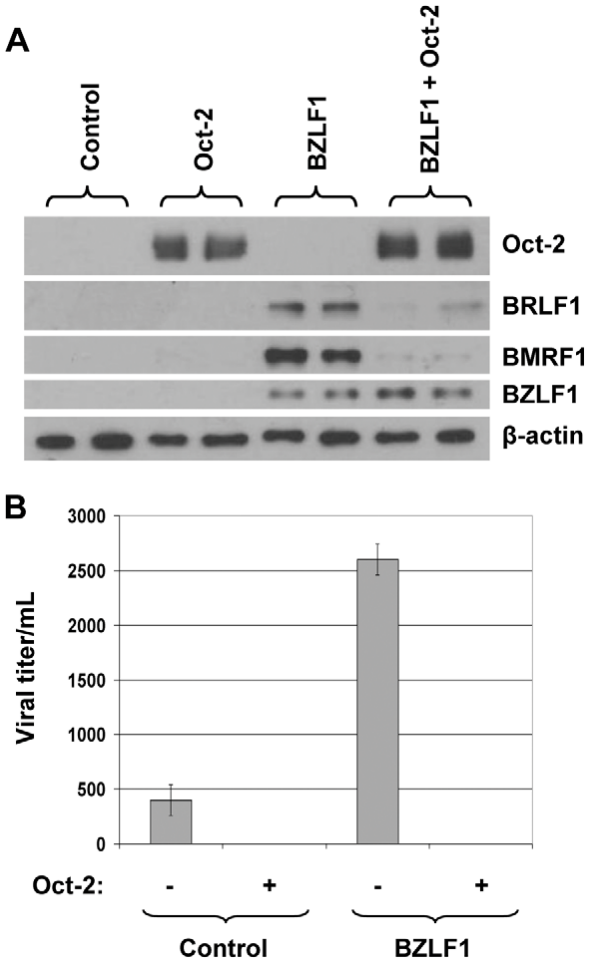
Oct-2 inhibits lytic EBV replication. (A) CNE-2 Akata cells were transfected with 5 ng BZLF1, 500 ng Oct-2, or control expression vectors as indicated. Immunoblot analysis was performed to compare the levels of transfected Oct-2 and BZLF1, as well as the lytic viral proteins BMRF1 and BRLF1 derived from the endogenous viral genome. β-actin expression was used as a loading control. (B) The amount of infectious virus produced by each condition in Figure 2A was determined using the green Raji cell assay as described in the Materials and Methods.

To examine whether Oct-2 also decreases BZLF1-initiated viral replication, CNE-2 Akata NPC cells were transfected with BZLF1 in the presence or absence of Oct-2. Transfected cell pellets were examined by immunoblot analysis to determine the effect of Oct-2 on lytic protein expression in the CNE-2 Akata cells (Figure 2A), and the amount of infectious viral particles released into the supernatant (from the same transfection) was quantitated three days after transfection using the green Raji cell assay (Figure 2B). CNE-2 Akata cells are stably infected with a GFP-expressing EBV that can be used to titer virus production.

Similar to the results in HONE-Akata cells, co-transfected Oct-2 inhibited the ability of transfected BZLF1 to induce lytic gene expression in CNE-2 Akata cells. Furthermore, transfected Oct-2 inhibited the amount of infectious virus produced from BZLF1-transfected cells, as well as the level of constitutive virus production. These results indicate that Oct-2 inhibits EBV lytic viral replication.

### Oct-2 inhibits BZLF1 activation of multiple lytic EBV promoters in EBV-negative cells

**Figure 3:**
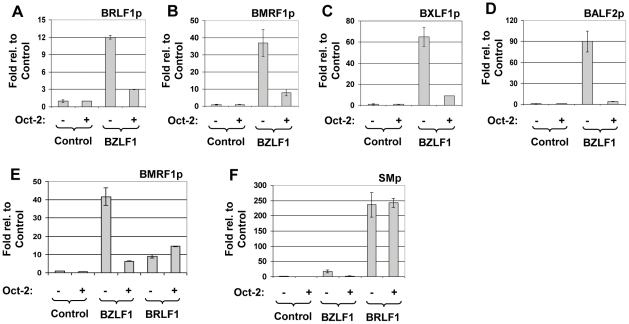
Oct-2 inhibits BZLF1 activation of multiple lytic EBV promoters in EBV-negative cells. EBV-negative HONE-1 cells were transfected with (A) BRLF1p-luciferase construct, (B) BMRF1p-luciferase construct, (C) BXLF1p-luciferase construct, or (D) BALF2p-luciferase construct in the presence or absence of co-transfected BZLF1 (5 ng), Oct-2 (100 ng), or control vectors as indicated. The fold luciferase activity for each condition is shown relative to control vector; the value for the activity of the promoter construct plus the vector control is set at 1. Values are given as means ± standard deviations of results from two replicates. EBV-negative HONE-1 cells were transfected with the (E) BMRF1p-luciferase construct or (F) SMp-luciferase construct in the presence or absence of co-transfected BZLF1 (5 ng), BRLF1 (5 ng), Oct-2 (100 ng), or control vectors as indicted.

Since the two EBV IE proteins, BZLF1 and BRLF1, activate one another's promoters, and cooperate to turn on expression of many early viral genes, it is difficult to distinguish between the effects of Oct-2 on BZLF1- versus BRLF1-mediated transcriptional function in the context of the intact viral genome. We therefore compared the effect of Oct-2 on BZLF1- versus BRLF1-mediated activation of early lytic EBV promoters using reporter gene assays in EBV-negative HONE-1 NPC cells. As shown in Figures 3A–F, co-transfected Oct-2 reduced BZLF1-mediated activation of each of the five different early lytic EBV promoters examined. In contrast, Oct-2 did not affect BRLF1-mediated activation of two different lytic viral promoters (Figures 3E–F). These results indicate that Oct-2 inhibits BZLF1, but not BRLF1, activation of lytic EBV promoters, and demonstrate that this effect is independent of any other viral proteins.

### Oct-2 inhibits BZLF1 DNA-binding *in vivo*

**Figure 4:**
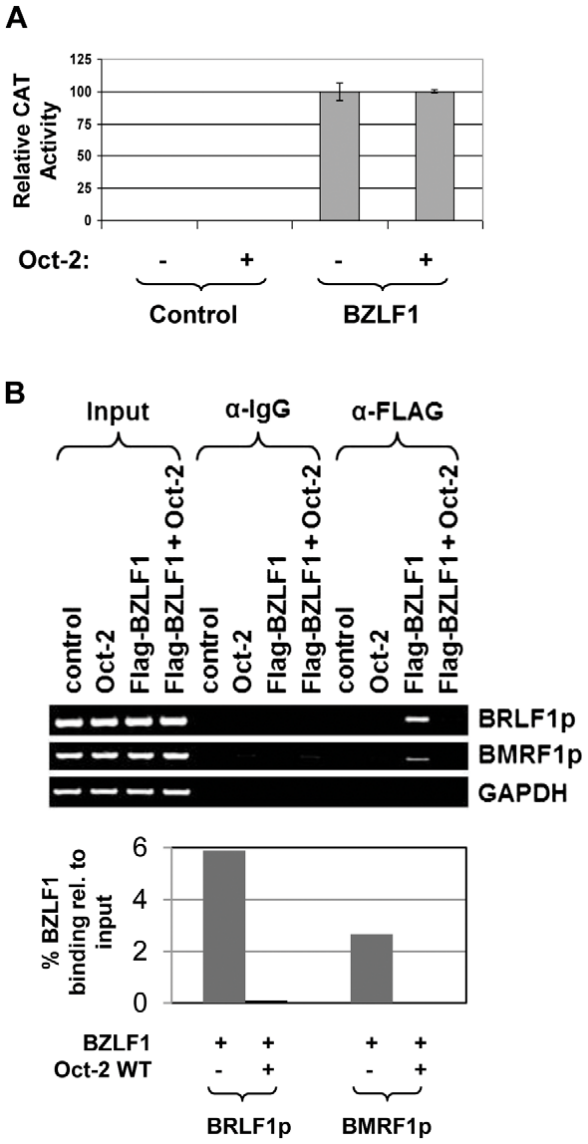
Oct-2 inhibits BZLF1 DNA-binding *in vivo*. (A) HeLa cells were transfected with a Gal4-E1B-CAT construct in the presence or absence of cotransfected Gal4-BZLF1 (1–167), Oct-2, or control vectors. The relative CAT activity for each condition is shown; the value for the activity of the promoter construct plus BZLF1 is set at 100. Values are given as means ± standard deviations of results from two independent experiments. (B) (Upper panel) A ChIP assay was performed using HONE-Akata cells transfected with Flag-tagged-BZLF1, Oct-2, or control vectors as indicated. Cross-linked DNA-protein complexes were immunoprecipitated using antibodies against Flag (BZLF1) or an IgG control. Antibody-bound DNA sequences were then PCR-amplified using primers spanning the EBV BRLF1 and BMRF1 promoters, or the GAPDH gene (negative control). (Lower panel) Binding bands were quantified using ImageJ software and represented as numerical values in bar diagrams in the lower panel. The amount of BZLF1 binding to each promoter in the presence or absence of Oct-2 was compared to input.

BZLF1 is a bZIP protein which contains an N-terminal transcriptional activation domain and a C-terminal DNA-binding/dimerization domain [26], [37], [77]–[81]. To further examine the effect of Oct-2 on BZLF1 transcriptional function per se (independent of BZLF1 DNA-binding), we performed reporter gene assays using the pGal4-BZLF1 (1–167) construct, which contains the transcriptional activation domain of BZLF1 fused to the Gal4 DNA-binding domain. The pGal4-BZLF1 (1–167) vector was co-transfected with the pGal4-E1B-CAT vector (which contains five copies of the Gal4 binding motif upstream of the minimal E1B promoter and CAT reporter gene) in the presence or absence of Oct-2, and the amount of CAT activity was determined. In contrast to its effect on BZLF1-mediated activation of viral lytic promoters, Oct-2 had no effect on BZLF1 transcriptional function in this assay (Figure 4A). This result indicates that Oct-2 does not inhibit the transcriptional function of BZLF1 when its DNA-binding domain is replaced by the Gal4 DNA-binding domain, and suggests that Oct-2 may instead inhibit BZLF1 DNA-binding activity.

We next performed ChIP assays to determine if Oct-2 inhibits BZLF1 binding to lytic EBV gene promoters *in vivo*. HONE-Akata cells were transfected with a Flag-tagged BZLF1 expression vector, in the presence or absence of co-transfected Oct-2, and the amount of BZLF1 binding to lytic EBV promoters was examined. As shown in Figure 4B, co-transfected Oct-2 greatly decreased the amount BZLF1 complexed to the BRLF1 promoter, as well as the BMRF1 early promoter. These results suggest that Oct-2 inhibits BZLF1 binding to lytic EBV promoters *in vivo*.

### BZLF1 interacts directly with Oct-2 *in vitro* and *in vivo*

**Figure 5:**
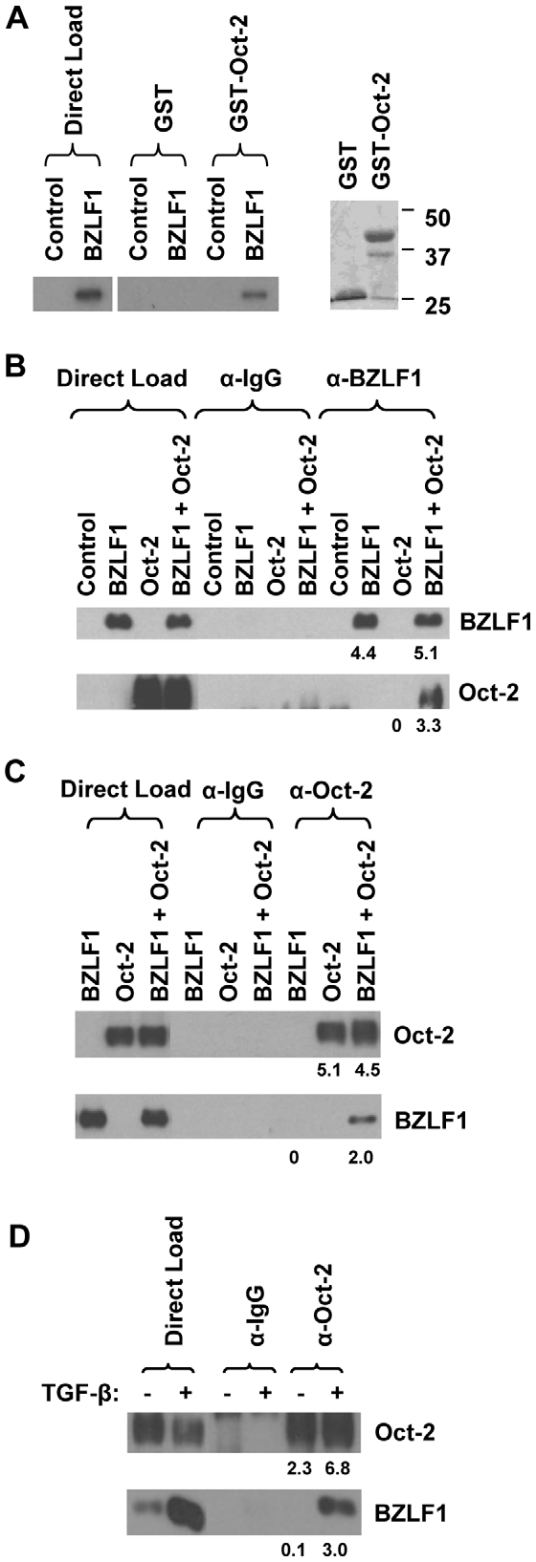
BZLF1 interacts directly with Oct-2 *in vitro* and *in vivo*. (A) (Left panel) GST pull-down assays were performed using GST or GST-Oct-2 fusion proteins incubated with ^35^S-labeled, *in vitro*-translated BZLF1 protein. Twenty percent of the direct load was used for autoradiography. (Right panel) A Coomassie stain demonstrating the levels of GST or GST-Oct-2 protein used in the GST pull-down assays. (B and C) EBV-negative HeLa cells were transfected with Oct-2 in the presence or absence of co-transfected BZLF1 and then immunoprecipitated with (B) a control mouse antibody or anti-BZLF1 mouse antibody or (C) a control rabbit antibody or anti-Oct-2 rabbit antibody. Five percent of the direct load was used for immunoblot analysis. Immunoprecipitated proteins were then examined by immunoblot analysis using anti-BZLF1 or anti-Oct-2 antibodies as indicated. The amount of BZLF1 and Oct-2 protein pulled down for each condition was quantitated relative to the corresponding input and is shown as a numerical value below each immunoblot; the amount of input for each condition was set at 100. (D) EBV-positive BL MutuI (type I latency) cells were treated with or without 5 µg/mL TGF-β for 24 hours and then immunoprecipitated with a control rabbit or Oct-2 rabbit antibody. Immunoprecipitated proteins were then examined by immunoblot analysis using anti-BZLF1 or anti-Oct-2 antibodies as indicated. Five percent of the direct load was used for immunoblot analysis. The amount of BZLF1 and Oct-2 protein pulled down for each condition was quantitated relative to the corresponding input and is shown as a numerical value below each immunoblot; the amount of input for each condition was set at 100.

To determine if Oct-2 interacts directly with BZLF1, we performed GST pull-down assays using *in vitro*-translated ^35^S-labeled BZLF1, and bacterially produced glutathione-S-transferase (GST) or GST-Oct-2 fusion proteins (containing only the Oct-2 POU domain, amino acids 179 to 343). Using this assay, we found that BZLF1 associates with GST-Oct-2, but not with the control GST protein (Figure 5A). To examine whether Oct-2 and BZLF1 can also interact when over-expressed *in vivo*, we transfected HeLa cells with BZLF1 and/or Oct-2 expression vectors and performed co-immunoprecipitation assays. As shown in Figures 5B and 5C, BZLF1 and Oct-2 can be co-immunoprecipitated when co-expressed in HeLa cells. To confirm that endogenous levels of BZLF1 and Oct-2 are able to interact in a B-cell environment, the EBV-positive BL cell line, MutuI, was treated with TGF-β to induce the lytic form of EBV infection, and cellular extracts were co-immunoprecipitated with anti-Oct-2 or control antibodies. BZLF1 was co-immunoprecipitated with endogenous Oct-2 in lytically infected MutuI cells (Figure 5D). Taken together, these results indicate that the BZLF1 and Oct-2 proteins directly interact *in vitro* and *in vivo*.

### Oct-2 interacts with BZLF1 DNA-binding/dimerization domain, and BZLF1 interacts with Oct-2 POU domain

**Figure 6:**
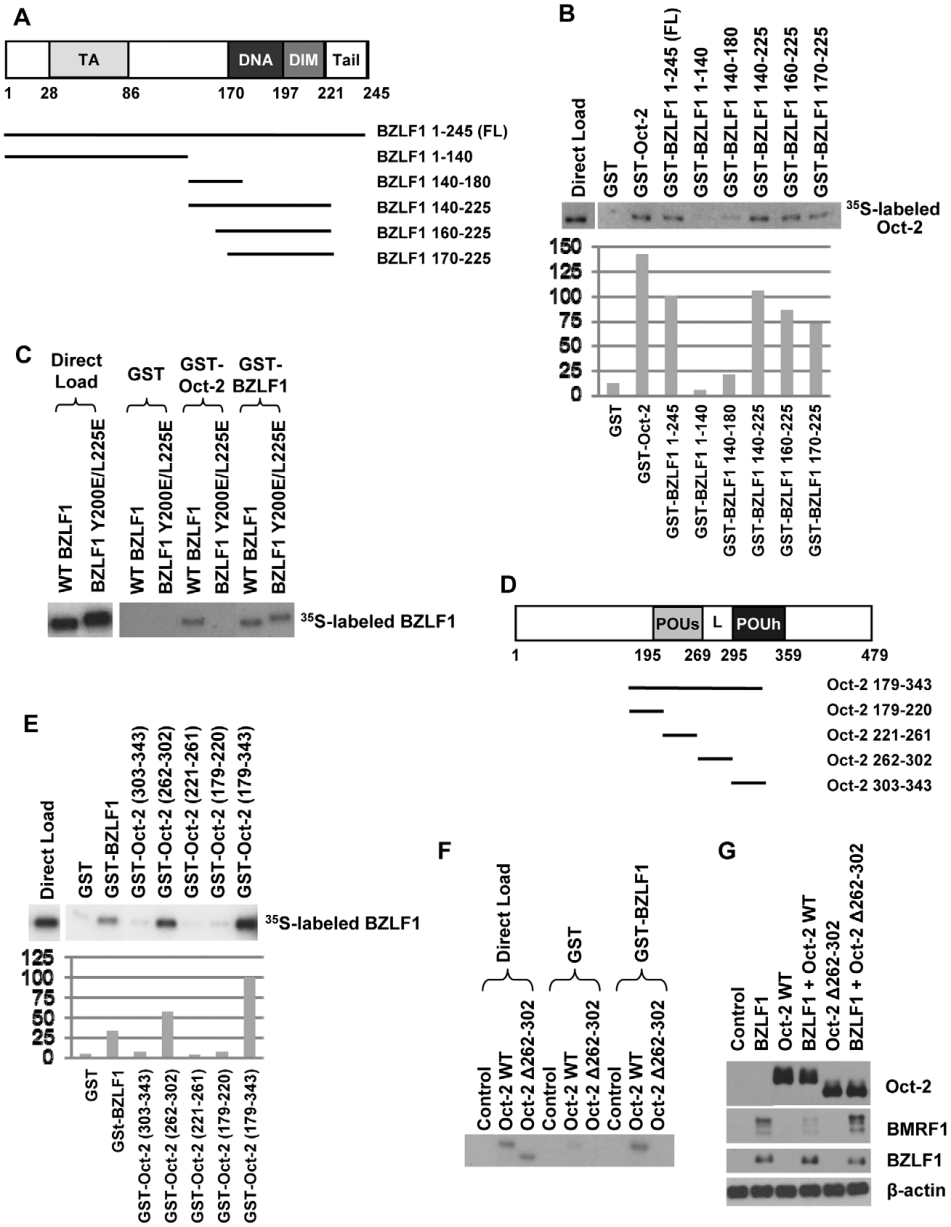
Oct-2 interacts with BZLF1 DNA-binding/dimerization domain, and BZLF1 interacts with Oct-2 POU domain. (A) Schematic of the BZLF1 protein transcriptional activation (TA), basic DNA-binding (DNA), dimerization (DIM), and C-terminal tail (Tail) domains. Numbers represent amino acid positions. Full-length (FL), as well as various BZLF1 truncation proteins used in subsequent GST pull-down assays are also depicted. (B) GST pull-down assays were performed using GST, GST-Oct-2, or various GST-BZLF1 truncation fusion proteins incubated with ^35^S-labeled, *in vitro*-translated Oct-2 protein. Twenty percent of the direct load was used for autoradiography. The amount of Oct-2 binding in each condition, quantified using ImageJ software, is depicted in a bar graph at the bottom of the gel; the level of binding obtained with the full-length (1–245) BZLF1 protein is set at 100 percent. (C) GST pull-down assays were performed using GST, GST-BZLF1, or GST-Oct-2 fusion proteins incubated with ^35^S-labeled, *in vitro*-translated wild-type BZLF1 or mutant BZLF1 (Y200E/L225E) protein. Twenty percent of the direct load was used for autoradiography. (D) Schematic of the Oct-2 protein POU specific domain (POUs), linker region (L), and POU homeodomain (POUh). Numbers represent amino acid positions. Various Oct-2 truncation proteins used in subsequent GST pull-down assays are also depicted. (E) GST pull-down assays were performed using GST, GST-BZLF1, or various GST-Oct-2 truncation fusion proteins incubated with ^35^S-labeled, *in vitro*-translated BZLF1 protein. Twenty percent of the direct load was used for autoradiography. The amount of BZLF1 binding in each condition, quantified using ImageJ software, is depicted in a bar graph at the bottom of the gel; the level of binding obtained with Oct-2 (179–343) protein is set at 100 percent. (F) GST pull-down assays were performed using GST or GST-BZLF1 fusion protein incubated with ^35^S-labeled, *in vitro*-translated (full-length) wild-type Oct-2 or mutant Oct-2 (Δ262–302) protein. Twenty percent of the direct load was used for autoradiography. (G) HONE-Akata cells were transfected with 5 ng BZLF1, 50 ng wild-type Oct-2, 50 ng mutant Oct-2 (Δ262–302), or control vectors as indicated. Immunoblot analysis was performed two days after transfection to compare levels of transfected BZLF1 and Oct-2, as well as the level of BMRF1 derived from the endogenous viral genome. β-actin served as a loading control.

To map the region of the BZLF1 protein required for the BZLF1/Oct-2 interaction, GST-BZLF1 deletions were constructed containing different portions of the BZLF1 protein fused to the GST moiety (as shown in Figure 6A), and GST pull-down assays were performed with *in vitro*-translated ^35^S-labeled Oct-2 protein. The results of these assays showed that Oct-2 associates with the DNA-binding/dimerization domain of BZLF1 (Figure 6B). To identify specific BZLF1 residues required for interaction with Oct-2, we also performed GST-Oct-2 pull-down assays using various ^35^S-labeled *in vitro* translated BZLF1 mutants that contain alterations in the DNA-binding/dimerization domain of BZLF1. As shown in Figure 6C, these assays identified a BZLF1 mutant, BZLF1 (Y200E/L225E), which is defective for interaction with the GST-Oct-2 protein, but retains the ability to interact with the GST-BZLF1 protein. Unfortunately, the BZLF1 (Y200E/L225E) mutant was found to be unstable when expressed *in vivo* (data not shown), and thus we could not determine if this mutant loses the ability to be inhibited by Oct-2 *in vivo*.

To further map the region of Oct-2 required for interaction with BZLF1, various GST-Oct-2 constructs were made which contain different portions of the Oct-2 POU domain (Figure 6D), and GST pull-down assays were performed with *in vitro*-translated ^35^S-labeled BZLF1 protein. The results of these assays indicate that Oct-2 amino acids 262 to 302 are sufficient to mediate the interaction with BZLF1 *in vitro* (Figure 6E). We next generated a mutant Oct-2 expression vector which has amino acids 262–302 deleted within the full-length Oct-2 protein. As shown in Figure 6F, this Oct-2 mutant is deficient for interaction with GST-BZLF1 *in vitro*.

To determine if the ability of Oct-2 to interact directly with BZLF1 is required for its ability to inhibit BZLF1 function *in vivo*, we compared the ability of the wild-type Oct-2 protein, versus the mutant Oct-2 (Δ262–302) protein, to inhibit BZLF1 function in the NPC cell line HONE-Akata. In contrast to wild-type Oct-2, the Oct-2 (Δ262–302) mutant was unable to inhibit BZLF1-mediated disruption of latency (Figure 6G).

### Oct-2 DNA-binding activity is not required for Oct-2 inhibition of BZLF1 function

**Figure 7:**
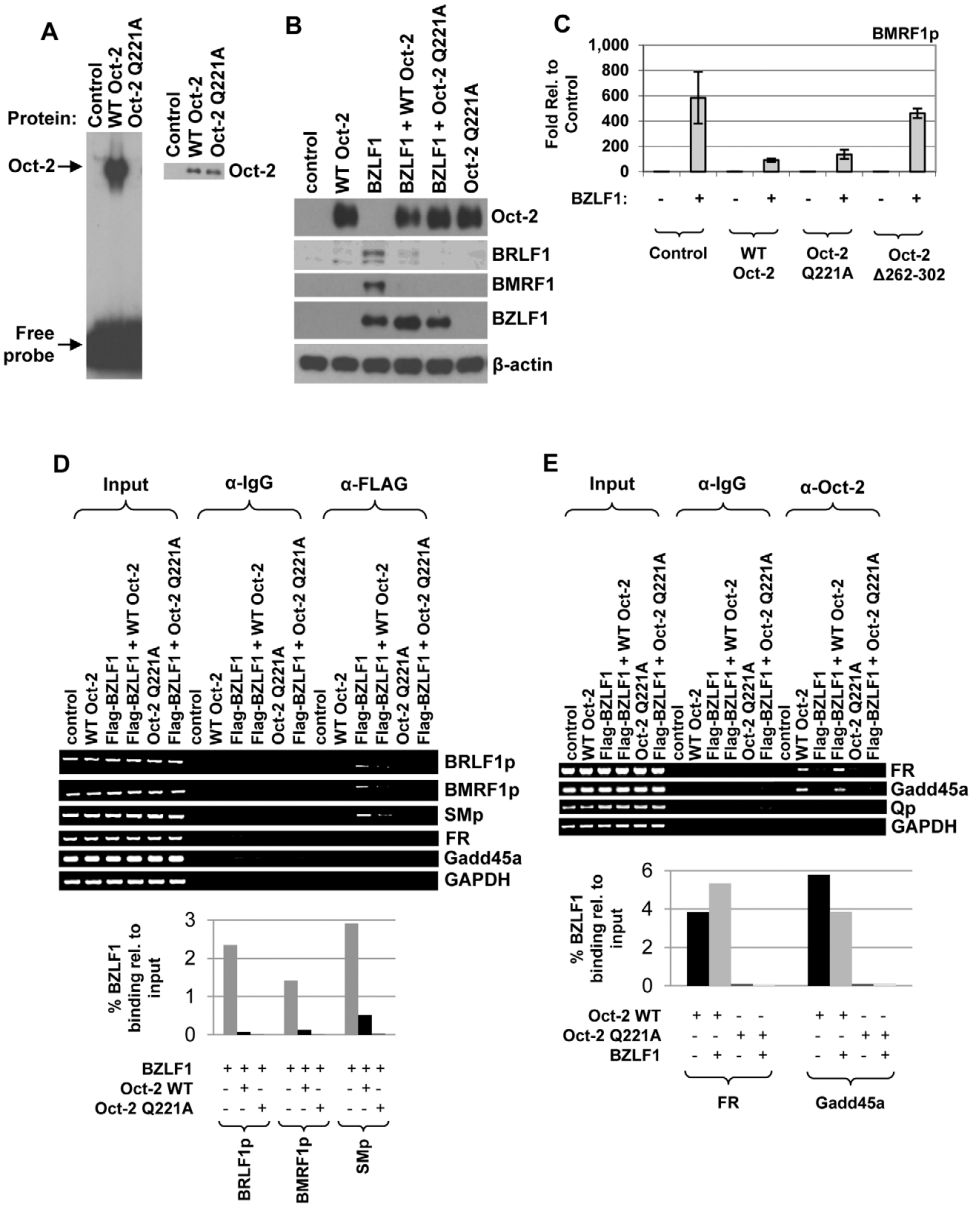
Oct-2 DNA-binding activity is not required for Oct-2 inhibition of BZLF1 function. (A) (Left panel) An EMSA was performed using *in vitro*-translated wild-type or mutant Oct-2 (Q221A) protein and a ^32^P-labelled oligonucleotide probe containing the consensus Oct-2 binding site. Protein-DNA complexes are indicated by arrows. (Right panel) Immunoblot analysis was performed to compare the levels of wild-type and mutant Oct-2 (Q221A) in the *in vitro*-translated samples. (B) HONE-Akata cells were transfected with 5 ng BZLF1, 500 ng wild-type Oct-2, 500 ng mutant Oct-2 (Q221A), or control vectors as indicated. Immunoblot analysis was performed two days after transfection to compare levels of transfected BZLF1 and Oct-2, as well as the levels of BMRF1 and BRLF1 protein derived from the endogenous viral genome. β-actin served as a loading control. (C) EBV-negative BJAB cells were transfected with a BMRF1p-luciferase construct in the presence or absence of co-transfected BZLF1 (30 ng), wild-type Oct-2 (1470 ng), two different mutant Oct-2 proteins (Oct-2 Q221A or Oct-2 Δ262–302) (1470 ng), or control vectors as indicated. The fold luciferase activity for each condition is shown relative to control vector; the value for the activity of the promoter construct plus the vector control is set at 1. Values are given as means ± standard deviations of results from two replicates. (D) A ChIP assay was performed using HONE-Akata cells transfected with Flag-tagged-BZLF1, wild-type Oct-2, mutant Oct-2 (Q221A), or control vectors as indicated. Cross-linked DNA-protein complexes were immunoprecipitated using antibodies against Flag (BZLF1) or an IgG control. Antibody-bound DNA sequences were then PCR-amplified using primers spanning the EBV BRLF1, BMRF1, or SM promoters, the viral FR repeats, cellular Gadd45a promoter, or the GAPDH gene (negative control). Binding bands were quantified using ImageJ software and represented as numerical values in bar diagrams in the lower panel. The amount of BZLF1 binding to each promoter in the presence or absence of wild-type or mutant Oct-2 (Q221A) was compared to input. (E) A ChIP assay was performed using HONE-Akata cells transfected with 3 µg wild-type Oct-2 or mutant Oct-2 (Q221A), with or without 3 µg co-transfected BZLF1, or control vectors as indicated. Cross-linked DNA-protein complexes were immunoprecipitated using antibodies against Oct-2 or an IgG control. Antibody-bound DNA sequences were then PCR-amplified using primers spanning the cellular Gadd45a promoter, viral FR repeats, viral latency Qp, or the GAPDH gene (negative control). Binding bands were quantified using ImageJ software and represented as numerical values in bar diagrams in the lower panel. The amount of Oct-2 binding to each DNA region in the presence or absence of BZLF1 was compared to input.

To examine the importance of Oct-2 DNA-binding activity on its ability to inhibit lytic EBV reactivation, we constructed an Oct-2 mutant altered at residue Q221, which was previously shown to be required for DNA-binding activity [82]. The Oct-2 mutant (Q221A) was shown to be unable to bind DNA *in vitro* (Figure 7A), and was stable when expressed *in vivo*.

We next compared the ability of the wild-type or DNA-binding defective mutant Oct-2 (Q221A) to inhibit BZLF1 function. Interestingly, the Oct-2 (Q221A) mutant was similar to the wild-type Oct-2 protein in regard to its ability to prevent BZLF1-mediated lytic reactivation in HONE-Akata cells (Figure 7B). The Oct-2 (Q221A) mutant was likewise similar to the wild-type Oct-2 protein in its ability to inhibit BZLF1 activation of the lytic EBV BMRF1 promoter in a reporter gene assay (performed in the EBV-negative B-cell line, BJAB) (Figure 7C), although the Oct-2 (Δ262–302) mutant was defective (consistent with results shown in Figure 6G). Furthermore, ChIP assays performed in HONE-Akata cells showed that both the wild-type and mutant Oct-2 (Q221A) proteins inhibit BZLF1 binding to lytic EBV promoters (Figure 7D). Taken together, these results suggest that Oct-2 DNA-binding activity is not required for the ability of Oct-2 to inhibit BZLF1-mediated lytic reactivation and/or BZLF1 DNA-binding. Instead, our findings are consistent with a model in which a direct protein-protein interaction between Oct-2 and BZLF1 inhibits BZLF1 binding to DNA.

Using ChIP assays, we also examined whether BZLF1 has an effect on Oct-2 DNA-binding (Figure 7E). Even when over-expressed at a high level (3 µg/10-cm dish), BZLF1 does not affect Oct-2 DNA-binding to either the cellular Gadd45a promoter or the EBV genome FR repeat element located upstream of the latency viral Cp [72]. These results also confirm that the mutant Oct-2 (Q221A) protein is defective for DNA-binding activity *in vivo*.

### Loss of endogenous Oct-2 increases constitutive and induced lytic gene expression in EBV-infected B cells

**Figure 8:**
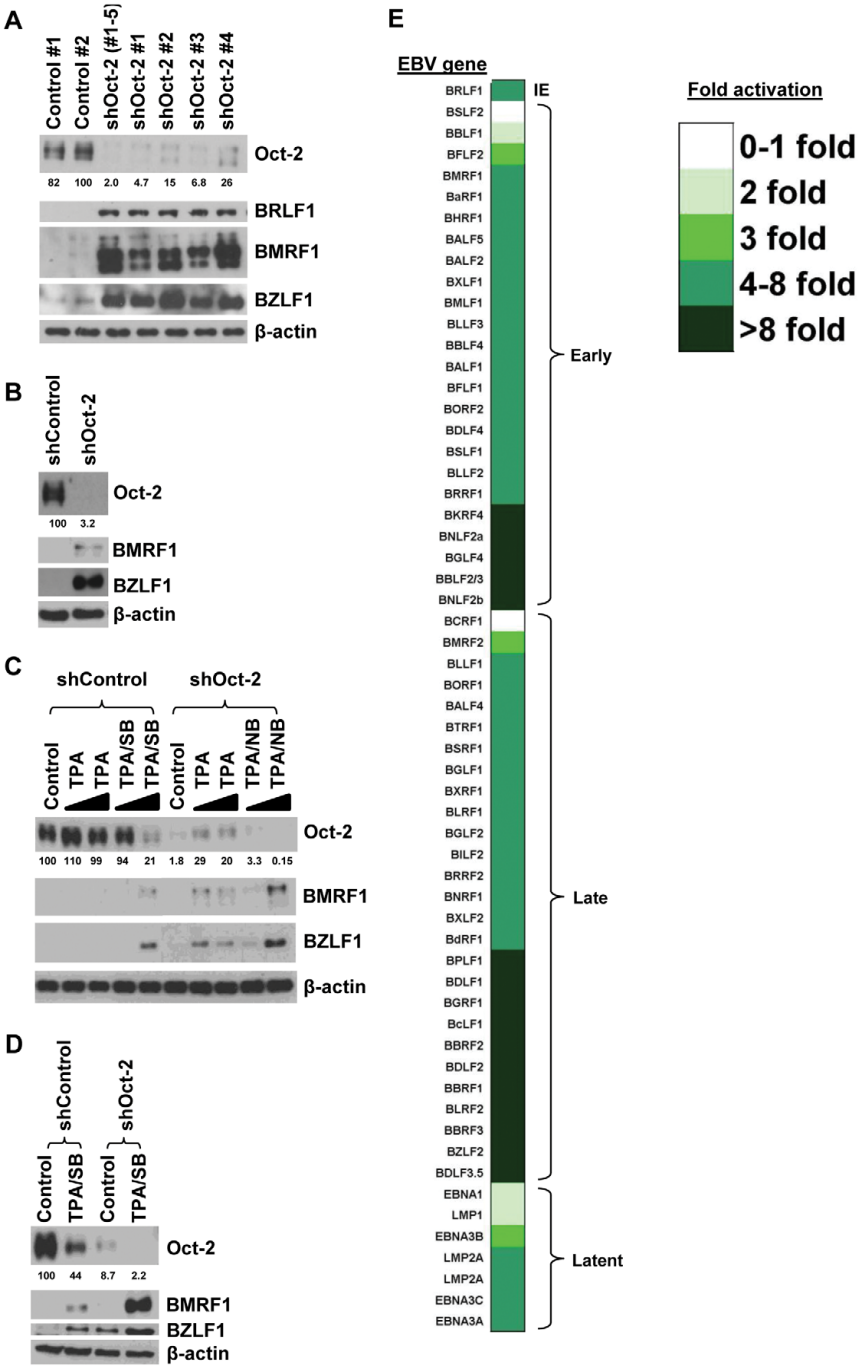
Loss of endogenous Oct-2 increases constitutive and induced lytic gene expression in EBV-infected B cells. (A) MutuI cells were infected with a pool of five different lentivirus vectors directed against Oct-2, individual lentivirus vectors directed against Oct-2, or control shRNAs. The cells were selected for 7 days using puromycin. Immunoblot analysis was performed to compare the levels of endogenous Oct-2, BRLF1, BZLF1, BMRF1, and β-actin (loading control) in each condition. The level of Oct-2 in each condition was quantitated relative to the Control #2 (set at 100) and is shown as a numerical value below the Oct-2 immunoblot. (B–D) KemI, Raji, and LCL cells were infected with a pool of five different lentivirus vectors directed against Oct-2 or control shRNAs and selected for 7 days using puromycin. The level of Oct-2 in each condition was quantitated relative to the untreated shControl (set at 100) and is shown as a numerical value below the Oct-2 immunoblot. (B) The type I BL cell line, KemI, was then subjected to immunoblot analysis to compare the levels of endogenous Oct-2, BZLF1, BMRF1, and β-actin (loading control) in both shOct-2 knockdown and control conditions. (C) Raji, a type III BL cell line, was treated with increasing amounts of TPA alone (4 or 20 ng/mL) or TPA (4 or 20 ng/mL) and sodium butyrate (0.6 or 3 mM) as indicated. Immunoblot analysis was performed after 24 hours to compare the levels of endogenous Oct-2, BZLF1, BMRF1, and β-actin (loading control) in each condition. (D) The type III lymphoblastoid cell line (LCL) was treated with 20 ng/mL TPA and 3 mM sodium butyrate for 48 hours to induce lytic reactivation, followed by immunoblot analysis for endogenous Oct-2, BZLF1, BMRF1, and β-actin (loading control). (E) RNA was isolated from control and shOct-2 infected MutuI cells, reverse-transcribed into cDNA, and analyzed for the level of viral gene expression using qRT-PCR as described in the Materials and Methods. Shown is a heatmap depicting the fold-activation of gene expression in the shOct-2 MutuI cells relative to the control vector infected cells. The EBV genes are grouped according to their gene expression profile (IE lytic, early lytic, late lytic, and latent). The fold gene activation is indicated by the color shade.

To examine the importance of endogenous Oct-2 expression on maintenance of EBV latency in B cells, we used shRNA vectors to knockdown Oct-2 expression in four different EBV-positive B-cell lines. As shown in Figure 8A, shRNA-mediated loss of endogenous Oct-2 expression in the type I BL cell line, MutuI (which has constitutive low level lytic gene expression), resulted in increased expression of three different lytic viral proteins: BZLF1, BRLF1, and BMRF1. Similar results were obtained using four different individual shRNAs directed against Oct-2. Knockdown of Oct-2 expression likewise increased lytic protein expression in another type I BL cell line, KemI (Figure 8B).

We also examined the effect of Oct-2 knockdown in two different EBV-positive B-cell lines with type III latency, Raji and LCL. In the Raji BL cell line (which has no detectable constitutive lytic viral protein expression, but can be induced to express lytic proteins following treatment with TPA/sodium butyrate), we found that loss of endogenous Oct-2 expression did not increase the level of constitutive lytic protein expression, but greatly increased the amount of lytic expression in response to TPA/sodium butyrate (Figure 8C). Similar results were obtained in an early passage lymphoblastoid cell line (LCL) (Figure 8D). These results indicate that Oct-2 acts as a negative regulator of lytic EBV gene expression when expressed at normal levels in B cells with either type I or III latency.

Since lytic reactivation is initiated by activation of the BZLF1 promoter, whereas the primary effect of Oct-2 appears to be mediated through its interaction with the BZLF1 protein, we also examined the effect of Oct-2 knockdown (or Oct-2 over-expression) on certain cellular transcription factors that have been previously shown to play important roles in regulating lytic reactivation through effects on BZLF1 promoter activity [41], [42], [46], [47]. As shown in Figure S1, we did not find that altering the level of Oct-2 in B cells (via Oct-2 knockdown) or in epithelial cells (via Oct-2 over-expression) had a consistent effect on the phosphorylation status of MEF-2D, the amount of ZEB1, or SMAD2 phosphorylation. These results suggest that Oct-2 does not induce lytic reactivation through additional effects on the BZLF1 promoter per se, and are consistent with the results in Figure 8 showing that Oct-2 knockdown alone is not sufficient to induce lytic reactivation in tightly latent B-cell lines such as Raji.

Finally, we used quantitative RT-PCR (qRT-PCR) to measure the effect of Oct-2 knockdown (in MutuI cells) on RNA levels of a wide variety of different EBV genes (Figure 8E). The results of this analysis indicate that following Oct-2 knockdown, the great majority of early and late lytic EBV genes have increased RNA expression (up to 20-fold elevated). Interestingly, as previously reported following anti-IgG mediated lytic induction of Akata cells [83], some viral latency gene transcripts were also increased following Oct-2 knockdown mediated lytic induction in MutuI cells (Figure 8E).

### Lytic reactivation stimuli decrease Oct-2 expression

**Figure 9:**
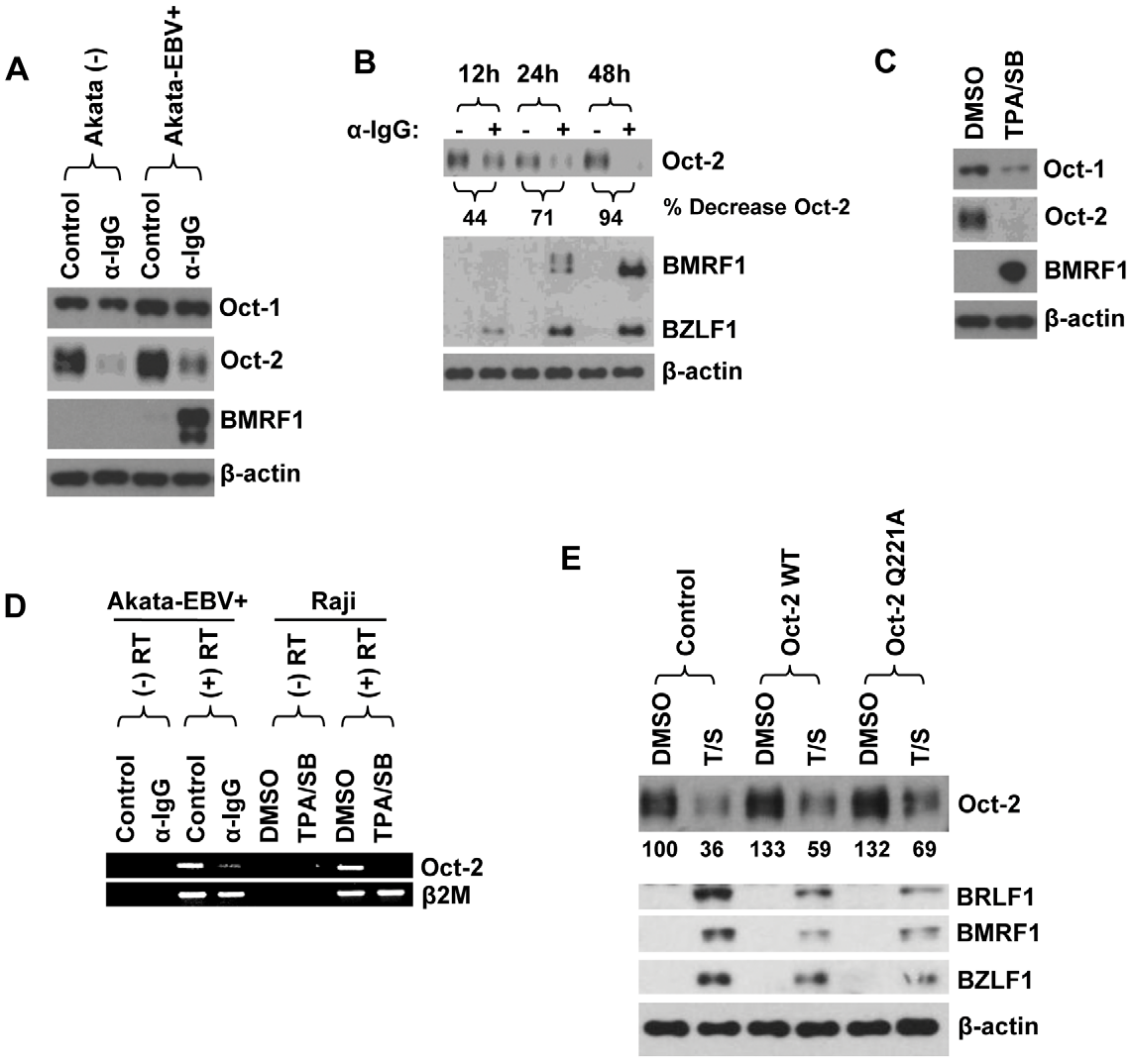
Lytic reactivation stimuli decrease Oct-2 expression. (A) The EBV-negative B-cell line, Akata (-), and the EBV-positive B-cell line, Akata-EBV+, were lytically induced with anti-IgG (20 µg/mL). Immunoblot analysis was performed two days after treatment to compare the levels of endogenous Oct-1, Oct-2, viral BMRF1 and β-actin (loading control). (B) Akata-EBV+ cells were lytically induced with anti-IgG (20 µg/mL) and harvested at 12, 24 and 48 hour intervals. Immunoblot analysis was performed to compare the levels of endogenous Oct-2, BMRF1, BZLF1, and β-actin (loading control). Oct-2 protein bands were quantified using ImageJ software and represented as percentage decrease of Oct-2 expression (relative to untreated cells at each time point) as shown below the Oct-2 immunoblot. (C) Raji cells were lytically induced with the chemical inducers TPA (20 ng/mL) and sodium butyrate (3 mM). Immunoblot analysis was performed two days after treatment to compare the levels of endogenous Oct-1, Oct-2, viral BMRF1 and β-actin (loading control). (D) The expression level of the Oct-2 gene was examined by RT-PCR in Akata-EBV+ and Raji cells lytically induced either with anti-IgG (20 µg/mL) or chemical inducers (20 ng/mL TPA/3 mM sodium butyrate) for 48 hours. The β_2_-microglobulin gene was used as a control. (E) Raji cells were infected with lentivirus vectors (CDH713) which express either wild-type Oct-2 or mutant Oct-2 (Q221A) proteins driven by the MSCV (murine stem cell virus) promoter and selected for 7 days using puromycin. Cells were then treated with 20 ng/mL TPA and 3 mM sodium butyrate for 24 hours to induce lytic reactivation. Immunoblot analysis was performed to compare the levels of Oct-2, BZLF1, BRLF1, BMRF1, and β-actin (loading control). The amount of Oct-2 protein expression was quantitated using ImageJ software and represented as a numerical value below the Oct-2 immunblot. The Oct-2 protein level in untreated cells infected with the control lentivirus was set at 100.

To determine if endogenous Oct-2 levels in B cells are affected by stimuli known to result in lytic EBV reactivation, we treated EBV-positive and EBV-negative BL Akata cells with or without anti-IgG (to induce cross-linking of the B-cell receptor). We found that the Oct-2 protein level is decreased in response to anti-IgG treatment in the presence or absence of the EBV genome (Figure 9A). Furthermore, the decrease in Oct-2 expression following anti-IgG treatment in EBV-positive Akata cells was apparent prior to the time point of maximal BMRF1 induction, suggesting that loss of Oct-2 contributes to BZLF1 activation of viral early genes (Figure 9B). We also examined the effect of TPA/sodium butyrate treatment on Oct-2 expression in the Type III BL cell line, Raji. As shown in Figure 9C, Oct-2 was decreased following TPA/sodium butyrate treatment.

To determine whether the decreased Oct-2 expression is due to impaired transcription versus a post-transcriptional mechanism, we examined the level of Oct-2 RNA transcripts in the Akata-EBV+ and Raji cells treated with or without lytic inducing stimuli (Figure 9D). Oct-2 RNA levels were greatly diminished by both types of lytic induction agents (anti-IgG and TPA/sodium butyrate), suggesting that loss of Oct-2 expression is likely mediated through a transcriptional mechanism.

Finally, to examine whether the loss of Oct-2 expression plays any role in the ability of TPA/sodium butyrate to induce lytic EBV gene expression in Raji cells, we stably infected Raji cells with control or Oct-2 expressing lentiviral vectors, in an attempt to restore Oct-2 expression in the TPA/butyrate treated cells. As shown in Figure 9E, cells infected with the WT or mutant Oct-2 (Q221A) lentiviral vectors expressed a higher level of Oct-2 than the vector control cells following treatment with TPA/sodium butyrate (presumably because the MSCV promoter driving Oct-2 expression in the lentiviral vector is resistant to the inhibitory effect), although full restoration of Oct-2 expression was not achieved. Nevertheless, even partial restoration of WT or mutant Oct-2 (Q221A) expression was sufficient to reduce the lytic induction effect of TPA/sodium butyrate (Figure 9E). Together, these results suggest that loss of Oct-2 expression may facilitate the ability of certain lytic inducing agents to reactivate lytic EBV gene expression in latently infected B cells.

### Oct-2 knockdown decreases EBNA1 protein expression in cells with type I and type III latency

**Figure 10:**
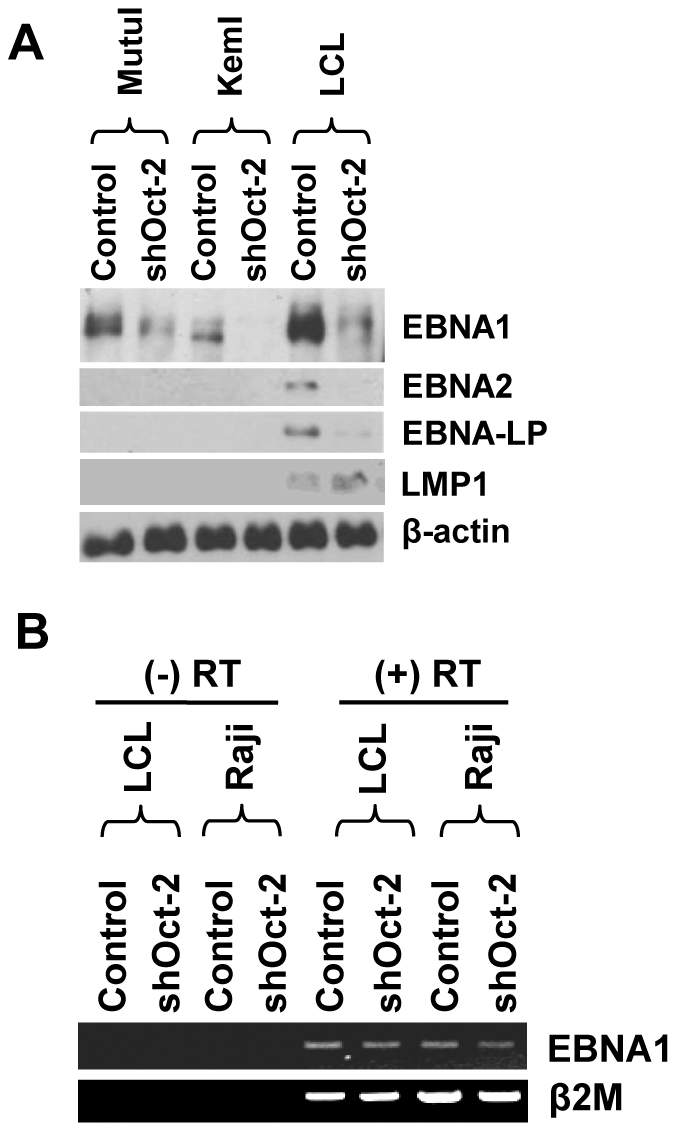
Oct-2 knockdown decreases EBNA1 protein expression in cells with type I and type III latency. (A) Type I BL cell lines, MutuI and KemI, and the type III lymphoblastoid cell line (LCL) were infected with a pool of five different lentivirus vectors directed against Oct-2, or control shRNAs. The cells were selected for 7 days using puromycin. The expression levels of the latency proteins EBNA1, EBNA2, EBNA-LP, and LMP1 were examined using immunoblot analysis. β-actin expression was used as a loading control. (B) The level of the EBNA1 transcript was examined by RT-PCR in type III LCL and Raji cells which were infected with a pool of five different lentivirus vectors directed against Oct-2, or control shRNAs. The cells were selected for 7 days using puromycin prior to RT-PCR analysis. The β_2_-microglobulin gene was used as a control.

Finally, since Oct-2 binding to the FR repeat element in the viral genome has been proposed to both decrease [74] or increase [72] the activity of the downstream Cp (which drives expression of the EBNA genes during type III latency), we examined the effect of Oct-2 knockdown on latent protein expression in type I versus type III EBV-positive cell lines. Interestingly, knockdown of Oct-2 reduced EBNA1 protein expression in cell lines with both type I and type III latency (Figure 10A). EBNA2 and EBNA-LP, which are also driven by the Cp , were also decreased following Oct-2 repression in type III cell lines, although LMP1 was not affected. However, the level of EBNA1 RNA was not decreased by loss of endogenous Oct-2 in cells with either type III (Figure 10B) or type I (Figure 8E) latency, suggesting that the Oct-2 effect on EBNA1 protein expression is primarily mediated through a post-transcriptional mechanism.

While loss of Oct-2 expression was not found to influence the type of viral latency, the finding that EBNA1 protein expression is dependent upon continued Oct-2 expression nevertheless provides further support for the notion that Oct-2 plays an essential role in promoting viral latency in B cells. Consistent with this notion, we found that long-term knockdown of Oct-2 in EBV-infected BL and LCL lines was incompatible with prolonged viability, and that such cell lines were dead within 14 days or had restored Oct-2 expression (data not shown). Together, these findings suggest that in addition to repressing lytic gene expression, Oct-2 may promote EBV latency by enhancing EBNA1 protein expression in both type I and type III latent infections.

## Discussion

The latent form of EBV infection ensures the long-term survival of the virus within the human host, and is an essential aspect of the viral life cycle. Although B cells are known to be the major site of viral latency [4], [6], the specific cellular transcription factor(s) that promote viral latency in a B-cell dependent manner have not been identified. In this report, we show that the B-cell specific transcription factor, Oct-2, promotes viral latency by directly interacting with, and inhibiting the function of, the viral BZLF1 IE protein.

Our results here show that Oct-2 inhibits BZLF1-mediated lytic reactivation in two different latently infected, EBV-positive NPC cell lines (HONE-Akata and CNE-2 Akata) (Figures 1 and 2). Oct-2 also abrogates both constitutive, and BZLF1-induced, lytic viral replication (Figure 2). Using reporter gene assays in EBV-negative cells, we demonstrate that Oct-2 inhibits BZLF1-mediated, but not BRLF1-mediated, activation of several different early lytic EBV promoters (Figure 3). Thus, the primary target of the Oct-2 inhibitory effect appears to be the BZLF1 protein.

To further explore the mechanism(s) by which Oct-2 reduces BZLF1-mediated activation, we examined the effect of Oct-2 on BZLF1 transcriptional function (using a construct in which the BZLF1 DNA-binding domain is replaced by the GAL4 DNA-binding domain), versus BZLF1 DNA-binding activity (Figure 4). Oct-2 was not found to affect BZLF1 transcriptional function per se. However, ChIP assays revealed that Oct-2 inhibits BZLF1 binding to early lytic EBV promoters *in vivo*.

We next asked if the Oct-2 and BZLF1 proteins directly interact. We indeed detected an interaction between the Oct-2 and BZLF1 proteins using both *in vivo* co-immunoprecipitation assays, as well as *in vitro* GST-fusion protein pull-down assays (Figure 5). Importantly, since we could also detect the interaction between endogenous BZLF1 and Oct-2 proteins in TGF-β treated MutuI cells (Figure 5), the Oct-2/BZLF1 interaction is not an artifact of over-expression systems. These results suggest that Oct-2 attenuates BZLF1 function by directly interacting with the BZLF1 protein and inhibiting its DNA-binding activity.

To further define the nature of the Oct-2/BZLF1 interaction, we mapped the regions of BZLF1 and Oct-2 required for this interaction (Figure 6). The region of BZLF1 encompassing its basic DNA-binding domain and the adjacent bZIP dimerization domain (residues 170 to 225) was found to be sufficient for BZLF1 interaction with Oct-2. In addition, our results showed that a 41 amino acid stretch (residues 262 to 302) within the POU domain of Oct-2 is sufficient for its interaction with BZLF1. By using an Oct-2 mutant (Δ262–302) which lacks the region required to interact with BZLF1, we confirmed that a direct interaction between Oct-2 and BZLF1 is required for Oct-2 inhibition of BZLF1 transcriptional function.

The findings that Oct-2 inhibits BZLF1 DNA-binding activity, and that an Oct-2 mutant (Δ262–302) that is unable to interact with BZLF1 is unable to inhibit BZLF1-mediated lytic reactivation, suggest a model in which Oct-2 inhibits BZLF1 function by forming an Oct-2/BZLF1 complex that cannot bind to BZLF1-response elements in EBV lytic promoters. To gain further support for this model (and since we were unable to identify a stable BZLF1 mutant that is specifically defective for the Oct-2 interaction), we next determined whether the DNA-binding activity of Oct-2 is required for its ability to inhibit BZLF1 function. Using a DNA-binding defective mutant, Oct-2 (Q221A), we showed that Oct-2 DNA-binding activity is not required for its ability to inhibit BZLF1 function (Figure 7). This result strongly suggests that Oct-2 inhibits BZLF1 function through a direct protein-protein interaction, rather than by competing for DNA-binding sites and/or by activating transcription of another cellular protein.

In contrast, we found that BZLF1 does not affect Oct-2 DNA-binding to either a cellular promoter, Gadd45a, or to the FR repeats in the EBV genome. In addition, BZLF1 was not found complexed to Oct-2 responsive promoters in the presence of Oct-2. These results suggest that BZLF1 may not globally regulate the ability of Oct-2 to activate Oct-2-responsive genes. Somewhat surprisingly, few (if any) genes in the human genome have been shown to require Oct-2 for their expression. Thus dissecting the effect (if any) of BZLF1 on Oct-2 mediated transcription will require further study.

To determine whether endogenous Oct-2 expression contributes to viral latency in EBV-infected B cells, we used shRNA vectors to knockdown endogenous Oct-2 in three different BL lines (MutuI, KemI, and Raji) and an LCL line (Figure 8). Loss of endogenous Oct-2 expression greatly increased the level of constitutive lytic viral protein expression in two different BL lines with type I latency (MutuI and KemI), as well as the ability of TPA/sodium butyrate treatment to induce lytic viral protein expression in the type III LCL line and Raji cells (a BL line with type III latency). Loss of endogenous Oct-2 expression in MutuI cells also results in increased RNA levels of many early and late lytic viral genes. Importantly, these results confirm that Oct-2 promotes viral latency when expressed at normal levels in B cells in the context of the intact virus, and in cells containing either type I or type III latency.

Similar to our finding here that Oct-2 promotes EBV latency in B cells; Oct-2 was recently reported to promote viral latency of another human gammaherpesvirus, KSHV [70]. Interestingly, although both viruses use the B-cell specific Oct-2 transcription factor to achieve viral latency in B cells, the mechanisms by which Oct-2 promotes latency for each virus are quite distinct. While the Oct-2 effect on EBV appears to be primarily mediated through the direct interaction between BZLF1 and Oct-2, and does not require Oct-2 DNA-binding activity, the inhibitory effect of Oct-2 on KSHV lytic reactivation requires Oct-2 DNA-binding activity and is thought to be mediated by direct Oct-2 binding to the KSHV IE ORF50 promoter [70]. Likewise, although both KSHV and EBV use Oct-1 as a positive regulator of viral reactivation, the mechanism(s) for the Oct-1 effect are somewhat different for each virus [55], [59], [60]. Furthermore, both viruses use the XBP-1 transcription factor as a means to tie viral reactivation to plasma cell differentiation [44], [45], [84].

B-cell differentiation into plasma cells (which is associated with lytic viral reactivation) results in loss of Oct-2 expression [76]. Interestingly, we found that Oct-2 expression was also rather dramatically decreased following treatment of EBV-positive BL cell lines with two different lytic-inducing stimuli (stimulation of the B-cell receptor with anti-IgG and treatment of cells with TPA/sodium butyrate) (Figure 9). Partial re-expression of Oct-2 (via lentivirus vectors) during TPA/sodium butyrate treatment of Raji cells decreased the amount of induced lytic viral protein expression, consistent with a role of Oct-2 loss in lytic gene reactivation. Furthermore, the Oct-2 decrease following anti-IgG treatment occurs prior to the maximal induction of the EBV BMRF1 early gene. Our results also show that the mechanism(s) by which anti-IgG and TPA/sodium butyrate reduce endogenous Oct-2 expression appears to be at the RNA level. Since anti-IgG treatment produced a similar effect in EBV-negative and EBV-positive Akata cells, the reduced Oct-2 expression following this treatment does not require an EBV-encoded gene product. In any event, the finding that both anti-IgG and TPA/sodium butyrate reduce Oct-2 expression in EBV-infected B cells suggests that loss of Oct-2 expression is one mechanism by which these agents induce lytic reactivation in B cells, in addition to their ability to activate BZLF1 gene expression.

Oct-2 has been previously proposed to regulate the type of EBV latency through regulation of the viral Cp [75]. According to this model, Oct-2 acts as a repressor of the Cp when bound to the upstream element FR, and B cells with high Oct-2 expression are predicted to exhibit type I latency, whereas cells with low Oct-2 expression are predicted to have type III latency. However, another report suggested that Oct-2 binding to the FR elements activates the Cp [72]. Here we found that knockdown of Oct-2 expression in cells with both type I latency and type III latency decreases EBNA1 protein expression through an apparently largely post-transcriptional mechanism (Figure 8E and Figure 10). Since the results of the previous papers were based on Oct-2 over-expression assays using reporter gene constructs, rather than Oct-2 knockdown studies in the context of the endogenous genome, we believe our studies are more likely to reflect the true effect of Oct-2 on EBV latent protein expression. Nevertheless, it is likely that the cellular level of Oct-2 transcriptional co-repressors (such as TLE1/2) [74] versus co-activators (such as Bob-1) [72], as well as the methylation state of the viral Cp, may influence the effects of Oct-2 on EBNA1 transcription. Additional studies will be necessary to determine the exact mechanism(s) by which Oct-2 regulates EBNA1 RNA and protein expression during type I versus type III latency. It is unknown whether lytic viral proteins play any role in mediating the post-transcriptional loss of EBNA1 expression.

**Figure 11:**
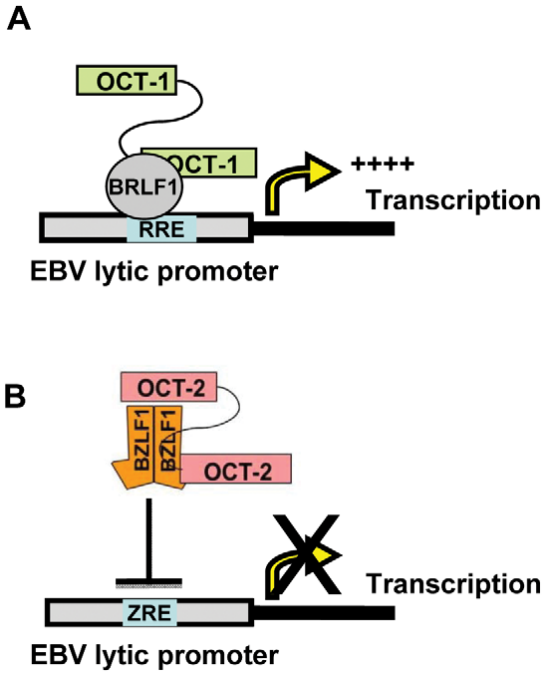
Hypothesized model for Oct-1 and Oct-2 regulation of EBV lytic reactivation. (A) Oct-1 enhances lytic reactivation through a direct protein-protein interaction between Oct-1 and the IE protein BRLF1, which promotes BRLF1 DNA-binding and tethers Oct-1 to viral DNA. (RRE; BRLF1 responsive element) (B) Oct-2 promotes latency by interacting with the BZLF1 IE protein and inhibiting its DNA-binding. (ZRE; BZLF1 responsive element).

In summary, our results here identify the B-cell specific cellular transcription factor, Oct-2, as being a potent negative regulator of EBV lytic reactivation. In contrast, our recent studies show that Oct-1 interacts with, and activates, the BRLF1 protein, which thereby promotes lytic viral reactivation. Our findings suggest a model in which the relative levels of Oct-1 versus Oct-2 influence whether EBV infection is latent versus lytic via their effects on BRLF1 and BZLF1 respectively (Figure 11). In addition, our findings suggest that Oct-2 may promote the establishment of EBV latency not only by inhibiting lytic gene expression, but also by increasing EBNA1 protein expression. We propose that Oct-2 plays a key role in allowing EBV to establish latency in a B-cell dependent manner.

## Materials and Methods

### Cell lines and culture

EBV-negative HeLa cells were maintained in DMEM supplemented with 10% fetal bovine serum (FBS) and 1% penicillin/streptomycin (pen/strep). EBV-negative HONE-1 cells (a gift from Ron Glaser, The Ohio State University) and BJAB cells (obtained from the ATCC) were maintained in RPMI 1640 supplemented with 10% FBS and 1% pen/strep. HONE-Akata (a gift from Lawrence Young, University of Birmingham) and CNE-2 Akata cells (a gift from K. W. Lo at The Chinese University of Hong Kong [received via Diane Hayward]) are nasopharyngeal carcinoma (NPC) epithelial cell lines super-infected with the Akata strain of EBV and were maintained in RPMI 1640 supplemented with 10% FBS, 1% pen/strep, and G418 (400 µg/mL). Both HONE-Akata and CNE-2 Akata cells are examples of type I latency cell lines. Akata (-) cells are an EBV-negative BL cell line that were maintained in RPMI 1640 supplemented with 10% FBS and 1% pen/strep. Akata-EBV+ cells (a gift from Kenzo Takada at Hokkaido University [received via Bill Sugden]) are a type I latency, BL cell line super-infected with the Akata strain of EBV and maintained in RPMI 1640 supplemented with 10% FBS, 1% pen/strep, and G418 (400 µg/mL). Raji cells (ATCC), an EBV-positive type III latency BL cell line, were maintained in RPMI 1640 supplemented with 10% FBS and 1% pen/strep. MutuI and KemI cells (gifts from Jeff Sample, Penn State) are EBV-positive type I latency BL cell lines and were maintained in RPMI 1640 supplemented with 10% FBS and 1% pen/strep. LCL cells, an early passage type III latency lymphoblastoid B-cell line transformed with the B95.8 strain of EBV, were maintained in RPMI 1640 supplemented with 10% FBS and 1% pen/strep. The cell type and viral latency status of the various cell lines used in this paper are summarized in Table S1.

### Plasmids, cloning, and site-directed mutagenesis

Plasmid DNA was purified on maxi-prep columns according to the manufacturer's protocol (Qiagen). pSG5 was obtained from Stratagene. The SG5-BRLF1 expression vector, a gift from S.D. Hayward, Johns Hopkins University, contains the genomic BRLF1 downstream of the simian virus 40 (SV40) promoter in the pSG5 vector. BZLF1 cDNA (a gift from Paul Farrell, Imperial College London) was cloned into the pSG5 vector to create pSG5-BZLF1 cDNA, which was also used to *in vitro* translate the BZLF1 protein. Flag-tagged-BZLF1, a gift from Paul Lieberman (Wistar Institute) has BZLF1 cDNA inserted into a p3XFLAG-myc-CMV24 vector (Sigma) for mammalian cell expression of a Flag-tagged-BZLF1 protein. The pSG5-Oct-2 expression vector was cloned by excising Oct-2 from pCGN-Oct-2 (a gift from Winship Herr, Cold Spring Harbor Laboratory) [85] and inserting it into a modified pSG5 vector (a gift from S.D. Hayward, Johns Hopkins University) using XbaI and BglII restriction sites. Plasmids pSG5-BZLF1 Y200E/L225E and pSG5-Oct-2 Q221A were constructed by using Stratagene QuikChange II XL Site-Directed Mutagenesis Kit and the following primer sets: BZLF1 Y200E forward 5′-GCCAAGTTTAAGCAACTGCTGCAGCACGAGCGTGAGGTCGCTGCTGCC-3′ and reverse 5′-GGCAGCAGCGACCTCACGCTCGTGCTGCAGCAGTTGCTTAAACTTGGC-3′; BZLF1 L225E forward 5′-GCAGATGTGCCCAAGCGAGGATGTTGACTCC-3′ and reverse 5′-GGAGTCAACATCCTCGCTTGGGCACATCTGC-3′; Oct-2 Q221A forward 5′-GCATCAAGCTGGGCTTCACGGCGGGTGATGTGGGCCTGG-3′ and reverse 5′-CCAGGCCCACATCACCCGCCGTGAAGCCCAGCTTGATGC-3′. pSG5-Oct-2 Δ262–302 was generated using overlapping PCR as described previously [86] using the following primer sets: Oct-2 deletion primer A 5′- CCGCGTCTAGAATGGGGGCTCCAGAAATAAG-3′, Oct-2 deletion primer B 5′-GGCTTCTGGTTCGCGCTTGAGTCCAC-3′, Oct-2 deletion primer C 5′- GTGGACTCAAGCGCGAACCAGAAGCC-3′, and Oct-2 deletion primer D 5′- CTGAGGGATCCTCAAGGCTGGTAAGGGGC-3′. All Oct-2 expression vectors contain the major B-cell form of Oct-2, isoform 1.

pGEX-KG and pGST-Oct-2 were a gift from Eric Turner (University of California-San Diego) [87]. pGST-BZLF1 was constructed by inserting BZLF1 amino acids (aa) 1-245 into pGEX-KG using SalI and SacI restriction sites. pGST-Oct-2 mutants were created by inserting Oct-2 aa179–220, aa221–261, aa262–302, or aa303–343 into pGEX-KG using SalI and HindIII restriction sites. pGST-BZLF1 1–140 was created by inserting BZLF1 aa1–140 into pGEX-KG using SalI and SacI restriction sites. All other pGST-BZLF1 mutants (140–180, 180–225, 140–225, 160–225, and 170–225) were created by inserting the corresponding BZLF1 amino acid sequences into pGEX-KG using SalI and HindIII restriction sites. Oct-2 WT and Oct-2 Q221A were cloned into the BamHI and EcoRI sites of the pCDH713 lentiviral vector (SBI, Cat# CD713B-1-SBI), under the control of the murine stem cell virus (MSCV) promoter, to create CDH713-Oct-2 WT and CDH713-Oct-2 Q221A.

The BRLF1p-luciferase reporter gene construct contains the Akata strain BRLF1p sequence from −1069 to +37 (relative to the BRLF1 transcription start site) inserted upstream of the luciferase gene in pGL3-basic (Promega) and was constructed as previously described [44]. The SMp, BMRF1p, and BALF2p-luciferase reporter gene constructs contain the B95.8 strain sequence from −595 to +15, −553 to +16, and −593 to +7 respectively (relative to transcription start site) inserted upstream of the luciferase gene in pGL3-basic using MluI and BglII restriction sites. The BXLF1p-luciferase reporter gene construct contains the BXLF1 promoter sequence (from 144859 to 145545) from the EBV B95.8 genomic DNA inserted upstream of the luciferase gene in pGL3-basic. The pGal4-BZLF1 (1–167) contains BZLF1 amino acids 1–167 inserted into pSG424 (M. Green, University of Massachusetts Medical Center), which contains the Gal4 DNA-binding domain. pGal4-E1B-CAT (M. Green, University of Massachusetts Medical Center) contains five copies of the Gal4 binding motif upstream of the E1B minimal TATA promoter and CAT (chloramphenicol acetyltransferase) reporter gene.

### Glutathione S-transferase pulldown assays

GST expression vectors were propagated in DH5α *E. coli* overnight. Cultures were diluted 1∶10, grown 2 hours and then induced using 0.4 mM IPTG for an additional 2 h. GST proteins were collected by sonication followed by incubation with glutathione-agarose beads (Sigma-Aldrich), rocking for 1 hour at room temperature. The beads were washed 3 times in GST buffer (20 mM HEPES [pH 7.7], 25 mM NaCl, 2.5 mM MgCl_2_, 0.1 mM EDTA, 1 mM dithiothreitol [DTT], 0.05% NP-40, protease inhibitor complete) and added to ^35^S-labelled *in vitro* translated protein. *In vitro*-translated proteins were generated using TNT T7 Quick Coupled Transcription/Translation System (Promega) in accordance with the manufacturer's instructions. The reaction mixture was incubated for 20 minutes with rocking at room temperature. The beads were washed 3 times in GST buffer. An equal volume of 2× SDS-sample buffer was added and proteins were extracted by heating at 95°C for 10 minutes.

### Immunoprecipitation

HeLa cells were transiently transfected with BZLF1 and/or Oct-2 expression vectors and then harvested 48 hours later. Alternatively, MutuI cells were treated with 5 µg/mL TGF-β (R&D Systems) for 48 hours. Cells were washed with 1× PBS and then incubated on ice with occasional rocking for 30 minutes in NP-40 lysis buffer (150 mM NaCl, 1% NP-40, 50 mM Tris [pH 8], and protease inhibitors). Cells were scraped into microcentrifuge tubes, sonicated for 15 s, and then centrifuged at maximum speed for 10 minutes at 4°C. Normal rabbit serum was added to the supernatant and incubated on ice 1 h. Protein A/G PLUS agarose beads (sc-2003; Santa Cruz Biotechnology) were added to preclear and the samples were rocked for an additional hour at 4°C. Beads were spun down, and the supernatant was divided for the appropriate conditions. 1 µg of antibody (or no antibody for the direct load) was added to each sample and rocked at 4°C for 1 h. The antibodies used were as follows: mouse anti-BZLF1 (sc-53904; Santa Cruz), rabbit anti-Oct-2 (sc-233; Santa Cruz Biotechnology), control mouse IgG (sc-2025), and control rabbit IgG (sc-2027). A/G beads were added and rocked at 4°C for 2 h. Beads were spun down and washed three times in NP-40 lysis buffer. An equal volume of 2× SDS-sample buffer was added and proteins were extracted by heating at 95°C for 10 minutes.

### Transient transfections

HONE-Akata cells were transfected using Lipofectamine 2000 Transfection Reagent (Invitrogen) according to the manufacturer's instructions. Each transfection experiment was performed at least three separate times with similar results. In general, cells were transfected in a 12-well dish with limiting amounts of BZLF1 or BRLF1 (5 ng), wild-type Oct-2 or mutant Oct-2 Q221A (500 ng), or control expression vectors (approximately 500 ng total DNA per well). In the case of the Oct-2 (Δ262–302) mutant studies, 5 ng of BZLF1 was cotransfected with or without 50 ng of wild-type or mutant Oct-2 proteins.

### Immunoblotting

Immunoblotting was performed as previously described [48], [88]. Cell lysates were harvested in SUMO lysis buffer containing proteasome inhibitor cocktail (Roche) and quantitated by SUMO Protein Assay (BioRad). Equivalent amounts of protein were separated in sodium dodecyl sulfate, 10% polyacrylamide gels and transferred to membranes. Membranes were blocked in PBS containing 5% milk and 0.1% Tween-20 solution and incubated with primary antibody. The following antibodies were used: anti-β-actin (Sigma; 1∶5000), BMRF1 (Vector; 1∶250), BRLF1 (Argene; 1∶250), BZLF1 (Santa Cruz, sc-53904; 1∶250), Oct-2 (Santa Cruz, sc-233; 1∶500), Oct-1 (Santa Cruz, sc-71744; 1∶250), EBNA1 (clone no. IH4 EBNA1 [89]; 1∶50), EBNA2 (Leica, clone no. PE2; 1∶100), EBNA-LP (clone no. JF186, 1∶100, gift from Paul Ling, Baylor), LMP1 (Dako, CS.1–4; 1∶100), MEF2D (Biosciences, 610774; 1∶10,000), ZEB1 (a gift from R. Burgess; 1∶250), and pSMAD2 (Ser465/467) (Cell Signaling; 1∶1,000). The secondary antibodies used were HRP goat-anti-mouse (Fisher Scientific; 1∶5,000), HRP goat-anti-rabbit (Fisher Scientific; 1∶10,000), and HRP donkey-anti-rat (Pierce; 1∶5,000)

### Virus lytic replication assays

Virus lytic replication titration assays were performed as previously described [90]. CNE-2 Akata cells were transfected using Lipofectamine 2000 Transfection Reagent (Invitrogen) in a 12-well dish with 5 ng BZLF1, 500 ng Oct-2, or vector controls. After 48 h, the cells were washed with 1× PBS and fresh RPMI media was added to the cells. After 24 h, supernatant from the transfected cells was collected and filtered through a 0.8-µm-pore-size filter. The filtered virus was used to infect Raji cells (4×10^5^ cells/infection). Phorbol-12-myristate-13-acetate (TPA; 20 ng/mL) and sodium butyrate (3 mM final concentration) were added at 24 hours post-infection. Green fluorescent protein (GFP)-positive Raji cells were counted 48 hours after infection to determine viral titer. Each condition was performed in duplicate.

### Reporter gene assays

All reporter gene constructs were methylated using *M. SssI* (NEB) according to the manufacturer's instructions and methylation was confirmed by digestion with the restriction enzyme HpaII (NEB), which cleaves its recognition sequence only if the DNA is not methylated at the cytosine residue within the CpG motif. HONE-1 cells were transfected using Lipofectamine 2000 Transfection Reagent (Invitrogen) in a 12-well dish with 50 ng luciferase construct, 5 ng BZLF1 or BRLF1, 100 ng Oct-2, or control expression vectors (500 ng total DNA per well). BJAB cells were also transfected using Lipofectamine 2000 Transfection Reagent in a 12-well dish with 100 ng luciferase construct, 30 ng BZLF1, 1470 ng Oct-2, or control expression vectors (1600 ng total DNA per well). Cells were harvested 48 hours post-transfection in Reporter Lysis 5× Buffer (Promega). Relative luciferase units were measured in a BD Monolight 3010 Luminometer (BD Biosciences) using Promega Luciferase Assay Reagent. Each condition was performed in duplicate. Extracts were also subjected to immunoblotting to verify equivalent protein levels.

### CAT assays

HeLa cells were transfected using FuGENE6 Transfection Reagent (Roche) according to the manufacturer's instructions in a 60 mm dish with 1 µg Gal4-E1B-CAT plasmid, 2.5 µg pGal4-BZLF1(1–167), 2.5 µg Oct-2, or control expression vectors (6 µg total DNA per dish). After 48 h, cells were harvested in ice cold 0.25 M Tris [pH 7.5] plus protease inhibitors (Roche) and subjected to freeze-thawing and centrifugation. The cell lysates were incubated at 37°C with acetyl coenzyme A and ^14^C-labeled chloramphenicol (Amersham Biosciences), as described previously [91]. The activity of the E1B promoter was measured by acetylation of chloramphenicol, and the percent acetylation was quantitated by thin-layer chromatography followed by autoradiography. The results were quantified using ImageQuant software (Amersham Biosciences). Extracts were also subjected to immunoblotting to verify equivalent protein levels.

### Chromatin immunoprecipitation (ChIP) assays

HONE-Akata cells were transfected in 10-cm dishes (0.5 µg Flag-tagged BZLF1, 5 µg Oct-2, or control vector). Cells were cross-linked in fresh 1% paraformaldehyde for 10 minutes at room temperature. The cross-linking reaction was quenched using 125 mM glycine. Following cell lysis and DNA fragmentation by sonication, DNA-protein complexes were immunoprecipitated with anti-FLAG (Sigma; F1804), anti-Oct-2 (Santa Cruz; sc-233), and control anti-IgG (Santa Cruz) antibodies. Immunoprecipitated DNA-protein complexes were washed using sequential low salt, high salt, lithium chloride and TE wash buffers. Protein-DNA cross-linking was reversed at 65°C overnight. DNA was purified using the Qiagen Gel Extraction Kit. The presence of specific DNA fragments in each precipitate was detected using PCR. Primers used for amplifying the SM promoter were 5′-CGTGACATGGAGAAACTGGGGG-3′ and 5′-CCTCTTACATCACTCACTGCACG-3′; BMRF1 promoter 5′-ATGCCCAGAAACCTGAGCAAGTAGCC-3′ and 5′-CCTTGGTGGATGTGCGAGCCATAAAG-3′; BRLF1 promoter 5′-CTCTTACCTGCGTCTGTTTGTG-3′ and 5′-CTCTCTGCTGCCCACTCATACT-3′; GAPDH 5′-TCACCACCATGGAGAAGGCT-3′ and 5′-GCCATCCACAGTCTTCTGGG-3′; Hs Gadd45a 5′-CTCCTCTCAACCTGACTCCAGGAG-3′ and 5′-TCCGGGGTTATCCTGCCAAC-3′; FR element 5′-GACTCTGCTTTCTGCCGTCT-3′ and 5′-CCTAACCATCCTTTTGCCAA-3′; Qp 5′- GACCACTGAGGGAGTGTTCCACAG -3′ and 5′-ACACCGTGCGAAAAGAAGCAC-3′.

### EMSA

T4 Polynucleotide Kinase (NEB) and gamma-^32^P-ATP (PerkinElmer) were used to label double-stranded, annealed DNA oligonucleotides for use in DNA-protein binding experiments. The Oct probe consisted of an 18-bp sequence containing the octamer consensus sequence (underlined) surrounded by random nucleotides (5′-CAGTGATGCAAATCTTGT-3′). The protein samples used in electrophoretic mobility shift assays (EMSAs) were *in vitro*-translated protein (made using TNT T7 Quick Coupled Transcription/Translation System [Promega]).

### Infection and packaging of lentivirus vectors

Lentiviral vectors expressing 5 different Oct-2 directed shRNAs (target set RHS4533), and the universal negative control, pLKO.1 (RHS4080) were purchased from Open Biosystems (ThermoScientific) and propagated according to the manufacturer's instructions. 293T cells were co-transfected with lentiviral vector(s) expressing shRNA, or Oct-2 proteins (pCD713-Oct-2 WT and Oct-2 mutant Q221A), plus DNA vectors encoding HIV Gag/Pol and VSV-G (for the packaging of lentiviruses) in 10-cm dishes. Media (containing lentivirus) was harvested 72 hours later and filtered through 0.8-µm-pore-size filters. MutuI, KemI, Raji and LCL cells were infected by incubation with filtered media containing the lentivirus. After 72 hours, stable cell lines were selected for 7 days by treatment with 1 µg/mL puromycin, at which time the various experiments were performed.

### RT-PCR

RNA was harvested from Akata-EBV+ cells (treated with or without anti-IgG treatment) and Raji cells (treated with or without TPA/sodium butyrate) after 48 hours using Qiagen RNeasy Mini Kit according to manufacturer's instructions. Isolated RNA was quantitated and DNase treated. Reverse transcriptase (RT)-PCR analysis was performed to determine the transcript levels of the endogenous cellular Oct-2 and β_2_-microglobulin genes. PCR primers used to detect Oct-2 transcript were 5′- GGCCCTCAACCTGAGCTTCAAG-3′ and 5′- GATCAGCAGGATCTCCTCT-3′; and β_2_-microglobulin transcript 5′-TTCTGGCCTGGAGGGCATCC-3′ and 5′-ATCTTCAAACCTCCATGATG-3′.

### Quantitative RT-PCR

Total RNA was isolated from 10∧6 cells per condition using Triazol (Sigma-Aldrich) as previously described [92]. mRNA was then enriched using Oligotex mRNA purification system (Qiagen) according to manufacturer's instructions. Reverse transcription was performed using the High Capacity cDNA Reverse Transcription Kit (ABI, cat# 4368814) according to manufacturer's instructions. EBV genome wide quantitative real-time PCR (qRT-PCR) was conducted as described previously at the UNC Vironomics Core Facility [93], [94]. All primers have a predicted Tm of 60±1°C and were purchased from MWG Operon Inc.. qRT-PCR was conducted on an LC480 LightCycler (Roche) under universal cycling conditions with 2× LightCycler 480 SYBR Green I Master qPCR mix (Roche) as the method of detection. The final primer concentration was 250 nM in a total 5 µl reaction. Data was collected in duplicate for each sample. The comparative Ct, or ΔΔCt method was used to measure the fold-changes in gene expression between samples.

## Supporting Information

Figure S1 **Effect of Oct-2 on MEF2D, SMAD2, and ZEB1.** Oct-2 levels were manipulated in B-cell lines (MutuI, KemI, LCL, and Raji) by infecting with a pool of five different lentivirus vectors directed against Oct-2, or control shRNAs. The cells were selected for 7 days using puromycin prior to immunoblot analysis. Oct-2 levels were also manipulated in the epithelial line, HONE-Akata (HA), by transfection with control vector or 500 ng of Oct-2 vector (500 ng DNA/12-well dish). (A) The KemI control and Oct-2 deficient B-cell lines, as well as the Oct-2 transfected HONE-Akata cells (HA), were examined by immunoblot for MEF2D phosphorylation using an antibody which recognizes total MEF2D. MEF2D phosphorylation was also examined in Akata-EBV+ cells induced for 48 hours with anti-IgG to serve as a positive control for MEF2D dephosphorylation. β-actin served as a loading control. (B) MutuI, KemI, LCL, and Raji control and Oct-2 deficient B-cell lines were examined by immunoblot for SMAD2 phosphorylation status using an antibody which recognizes phospho-SMAD2. MutuI cells treated with 5 ug/mL of TGF-β for 48 hours served as a positive control. β-actin served as a loading control. (C) Raji and KemI control and Oct-2 deficient B-cell lines, as well as HONE-Akata cells (HA) transfected with control vector or 500 ng of Oct-2 vector (500 ng DNA/12-well dish), were examined by immunoblot for ZEB1 expression. β-actin served as a loading control.
(TIF)

Table S1 **Cell types, EBV status, and EBV latency type of cell lines used in this study.**
(TIF)
