# Supplementary figures and images for: The B-Cell Specific Transcription Factor, Oct-2, Promotes Epstein-Barr Virus Latency by Inhibiting the Viral Immediate-Early Protein, BZLF1
Source: PLoS Pathog. 2012 Feb 9;8(2):e1002516. doi: 10.1371/journal.ppat.1002516 (PMC3276558; doi:10.1371/journal.ppat.1002516)

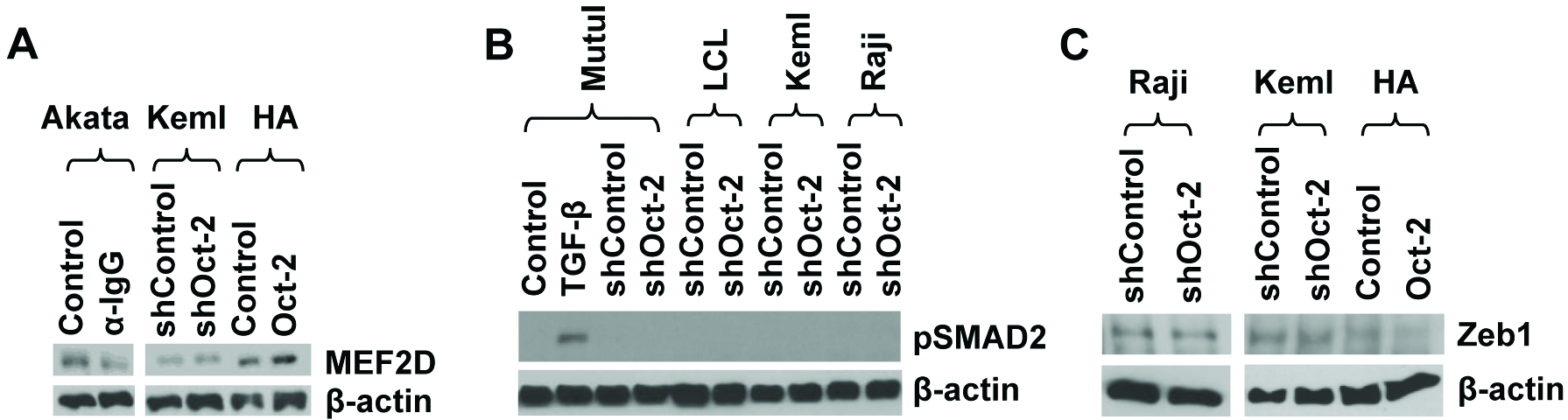

Supplement: Figure S1 — Effect of Oct-2 on MEF2D, SMAD2, and ZEB1. Oct-2 levels were manipulated in B-cell lines (MutuI, KemI, LCL, and Raji) by infecting with a pool of five different lentivirus vectors directed against Oct-2, or control shRNAs. The cells were selected for 7 days using puromycin prior to immunoblot analysis. Oct-2 levels were also manipulated in the epithelial line, HONE-Akata (HA), by transfection with control vector or 500 ng of Oct-2 vector (500 ng DNA/12-well dish). (A) The KemI control and Oct-2 deficient B-cell lines, as well as the Oct-2 transfected HONE-Akata cells (HA), were examined by immunoblot for MEF2D phosphorylation using an antibody which recognizes total MEF2D. MEF2D phosphorylation was also examined in Akata-EBV+ cells induced for 48 hours with anti-IgG to serve as a positive control for MEF2D dephosphorylation. β-actin served as a loading control. (B) MutuI, KemI, LCL, and Raji control and Oct-2 deficient B-cell lines were examined by immunoblot for SMAD2 phosphorylation status using an antibody which recognizes phospho-SMAD2. MutuI cells treated with 5 ug/mL of TGF-β for 48 hours served as a positive control. β-actin served as a loading control. (C) Raji and KemI control and Oct-2 deficient B-cell lines, as well as HONE-Akata cells (HA) transfected with control vector or 500 ng of Oct-2 vector (500 ng DNA/12-well dish), were examined by immunoblot for ZEB1 expression. β-actin served as a loading control. (TIF) [file ppat.1002516.s001.tif]
